# GUCY2C signaling limits dopaminergic neuron vulnerability to toxic insults

**DOI:** 10.1038/s41531-024-00697-z

**Published:** 2024-04-13

**Authors:** Lara Cheslow, Matthew Byrne, Jessica S. Kopenhaver, Lorraine Iacovitti, Richard J. Smeyne, Adam E. Snook, Scott A. Waldman

**Affiliations:** 1 Department of Pharmacology, Physiology, & Cancer Biology, Thomas Jefferson University, Philadelphia, PA USA; 2 Department of Neurosciences, Thomas Jefferson University, Philadelphia, PA USA; 3 Department of Microbiology & Immunology, Thomas Jefferson University, Philadelphia, PA USA; 4 Sidney Kimmel Cancer Center, Thomas Jefferson University, Philadelphia, PA USA

**Keywords:** Parkinson's disease, Cellular neuroscience

## Abstract

Mitochondrial dysfunction and reactive oxygen species (ROS) accumulation within the substantia nigra pars compacta (SNpc) are central drivers of dopaminergic (DA) neuron death in Parkinson’s disease (PD). Guanylyl cyclases and their second messenger cyclic (c)GMP support mitochondrial function, protecting against ROS and promoting cell survival in several tissues. However, the role of the guanylyl cyclase-cGMP axis in defining the vulnerability of DA neurons in the SNpc in PD remains unclear, in part due to the challenge of manipulating cGMP levels selectively in midbrain DA neurons. In that context, guanylyl cyclase C (GUCY2C), a receptor primarily expressed by intestinal epithelial cells, was discovered recently in midbrain DA neurons. Here, we demonstrate that GUCY2C promotes mitochondrial function, reducing oxidative stress and protecting DA neurons from degeneration in the 1-methyl-4-phenyl- 1,2,3,6-tetrahydropyridine (MPTP) mouse model. GUCY2C is overexpressed in the SNpc in PD patients and in mice treated with MPTP, possibly reflecting a protective response to oxidative stress. Moreover, cGMP signaling protects against oxidative stress, mitochondrial impairment, and cell death in cultured DA neurons. These observations reveal a previously unexpected role for the GUCY2C-cGMP signaling axis in controlling mitochondrial dysfunction and toxicity in SNpc DA neurons, highlighting the therapeutic potential of targeting DA neuron GUCY2C to prevent neurodegeneration in PD.

## Introduction

Parkinson’s disease (PD) is the second most common neurodegenerative disorder in the United States, affecting ≥1% of adults over 60 years of age^1,2^. Motor deficits, including resting tremor, cogwheel rigidity, and bradykinesia, reflect selective loss of dopaminergic (DA) neurons within the substantia nigra pars compacta (SNpc) and subsequent depletion of DA in the dorsal striatum^1–3^. Current PD treatments mitigate clinical symptoms by raising striatal DA levels (such as levodopa (l-DOPA), monoamine oxidase (MAO) inhibitors, and catecholamine-*O*-methyl- transferase inhibitors) or mimicking the effects of DA signaling in the striatum (such as DA agonists)^2–4^ but do not slow DA neurodegeneration and disease progression. Beyond the inability of PD medications to intervene in SNpc degeneration, there are no FDA-approved predictive biomarkers for detecting PD progression prior to SNpc neurodegeneration and the development of clinical symptoms^5^. Given the burden of disease, there is an urgent need to develop approaches for early detection and effective treatment of PD.

The etiology of PD is complex and multifaceted. Of PD cases, 10–15% are familial, and >20 gene mutations have been linked to PD development, including glucocerebrosidase (GBA), alpha-synuclein (SNCA), leucine-rich repeat kinase 2 (LRRK2), PTEN-induced kinase 1 (PINK1), parkin, and parkinsonism-associated deglycase (DJ-1, also known as PARK8)^1,6–9^. However, most PD cases are sporadic and have been closely correlated with environmental factors, including exposure to heavy metals, agricultural pesticides, and influenza infection^10–12^. A mechanistic link uniting the broad range of risk factors for PD development was discovered in 1982, when patients who inadvertently injected contaminated heroin presented to the clinic with l-DOPA-responsive PD-like symptoms. These patients had unknowingly consumed 1-methyl-4-phenyl- 1,2,3,6-tetrahydropyridine (MPTP), a lipophilic compound that crosses the blood-brain barrier (BBB) and is metabolized by MAO-B to its active form, 1-methyl-4-phenylpyridinium (MPP+)^13^. MPP+ is selectively taken up by DA transporter (DAT) expressed by DA neurons, and inhibits complexes I and IV of the mitochondrial electron transport chain (ETC), leading to an accumulation of reactive oxygen species (ROS) and apoptosis^14^. The discovery of MPTP as a causative agent of Parkinsonism introduced impaired mitochondria as a crucial driver of pathology. Despite the wide range of factors contributing to PD, impaired mitochondrial respiration and turnover and elevated levels of ROS within SNpc DA neurons are nearly ubiquitous among PD patients^15–18^.

Guanylyl cyclase C (GUCY2C) is a transmembrane receptor expressed in the intestinal epithelium, hypothalamus, ventral tegmental area (VTA), and nigrostriatal pathway^19,20^. Upon stimulation with exogenous ligands heat-stable enterotoxin (STa) or linaclotide (LIN), or endogenous intestinal hormones guanylin and uroguanylin, GUCY2C catalyzes the conversion of guanosine triphosphate (GTP) to cyclic guanosine monophosphate (cGMP)^21^. Mice from which GUCY2C protein expression has been eliminated [GUCY2C Knockout (KO), *Gucy2c*^−/−^ mice] have dysregulated mitochondria, elevated ROS, and oxidative damage in gut epithelia^22^, suggesting a potential parallel role for the receptor in other cells expressing GUCY2C beyond the intestine.

The present studies demonstrate that GUCY2C is expressed within midbrain DA neurons, and loss of GUCY2C increases ROS and reduces mitochondrial function in the nigrostriatal pathway. The absence of GUCY2C creates a vulnerability in SNpc, with DA neuron loss and gliosis following subtoxic doses of MPTP, suggesting that GUCY2C plays a protective homeostatic role in DA neurons. In that context, GUCY2C is increased in the SNpc of patients with PD and mice exposed to MPTP, suggesting a protective feedback loop between dysfunctional mitochondria associated with oxidative stress and GUCY2C-cGMP signaling. Indeed, cGMP enhances antioxidant capacity and cell survival, protecting against MPP+-induced ROS upregulation, loss of mitochondrial integrity, and cell death in DA neurons in vitro.

These observations reveal an unexpected role for the GUCY2C-cGMP signaling axis in regulating the toxic vulnerability of SNpc DA neurons, identifying GUCY2C as a possible therapeutic target for early intervention to protect against DA neuron degeneration. As GUCY2C is specifically expressed in DA and hypothalamic neurons within the CNS^20^, targeting GUCY2C to raise cGMP within DA neurons will have limited off-target effects. We also reveal a potential feedback loop identifying GUCY2C as a transcriptionally regulated target in response to oxidative stress in DA neurons. In addition to the therapeutic potential of targeting GUCY2C, this discovery suggests that GUCY2C may hold potential as a biomarker for early PD pathogenesis prior to significant DA neuron death.

## Results

### Functional GUCY2C is expressed by DA neurons within the SNpc

**Fig. 1:**
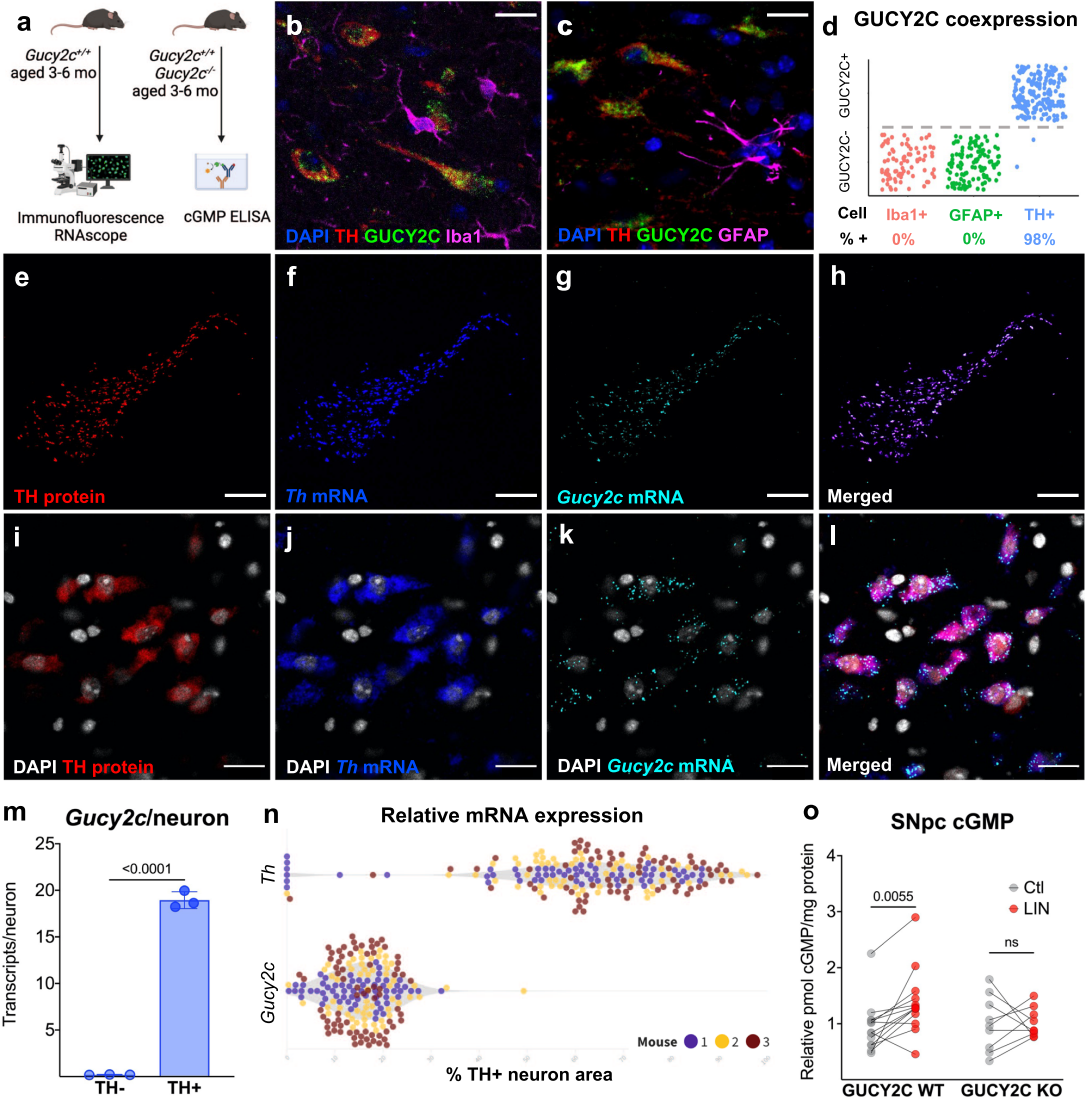
Functional GUCY2C protein and mRNA are expressed by DA neurons within the SNpc. **a** Schematic of mice used for analyses. **b**–**d** Immunofluorescence staining reveals that guanylyl cyclase C (GUCY2C) protein is expressed in 98% of tyrosine hydroxylase (TH)+ neurons, but not in astrocytes or microglia, in the mouse midbrain (*n* = 3). Scale bars represent 20 µM. **e**–**l** Combined immunofluorescence and RNAscope identifies high levels of *Gucy2c* mRNA co-expressed with TH protein and mRNA. Scale bars represent 200 µM (**e**–**h**) or 20 µM (**i**–**l**). **m** *Gucy2c* mRNA is not expressed by TH-negative cells (*n* = 3). **n** *Gucy2c* mRNA is expressed at nearly a third of *Th* mRNA levels in DA neurons (*n* = 3) as determined through RNAscope. **o** Treating *Gucy2c*^+/+^(WT), but not *Gucy2c*^−/−^(KO), SNpc with the GUCY2C agonist linaclotide (LIN), but not with inactive peptide control, upregulates intracellular cGMP production (*n* = 9–11). Statistics were calculated using a two-tailed *t*-test (**m**) or a two-way ANOVA with a false discovery rate <0.05 (**o**). Error bars displayed represent the standard error of the mean (SEM).

Previously, we demonstrated that GUCY2C is transcribed and translated in the SNpc prior to being trafficked to the striatum^20^. Here, we further characterized the specificity of GUCY2C protein and mRNA expression in the nigrostriatal pathway (Fig. 1a). GUCY2C protein (Fig. 1b–d) and mRNA (Fig. 1e–m) are expressed on 98% of TH+ neurons in the SNpc, but not on microglia or astrocytes.

GUCY2C mRNA is expressed at nearly a third of TH mRNA levels in DA neurons (Fig. 1n). The GUCY2C agonist LIN stimulates production of intracellular (Fig. 1o), but not extracellular (supplemental Fig. 1), cGMP in SNpc from wild type (WT; *Gucy2c*^+/+^), but not GUCY2C KO (*Gucy2c*^−/−^), mice demonstrating the utility of targeting SNpc GUCY2C to raise intracellular cGMP levels specifically in DA neurons.

### Loss of GUCY2C leads to mitochondrial dysfunction and oxidative stress within the nigrostriatal pathway

**Fig. 2:**
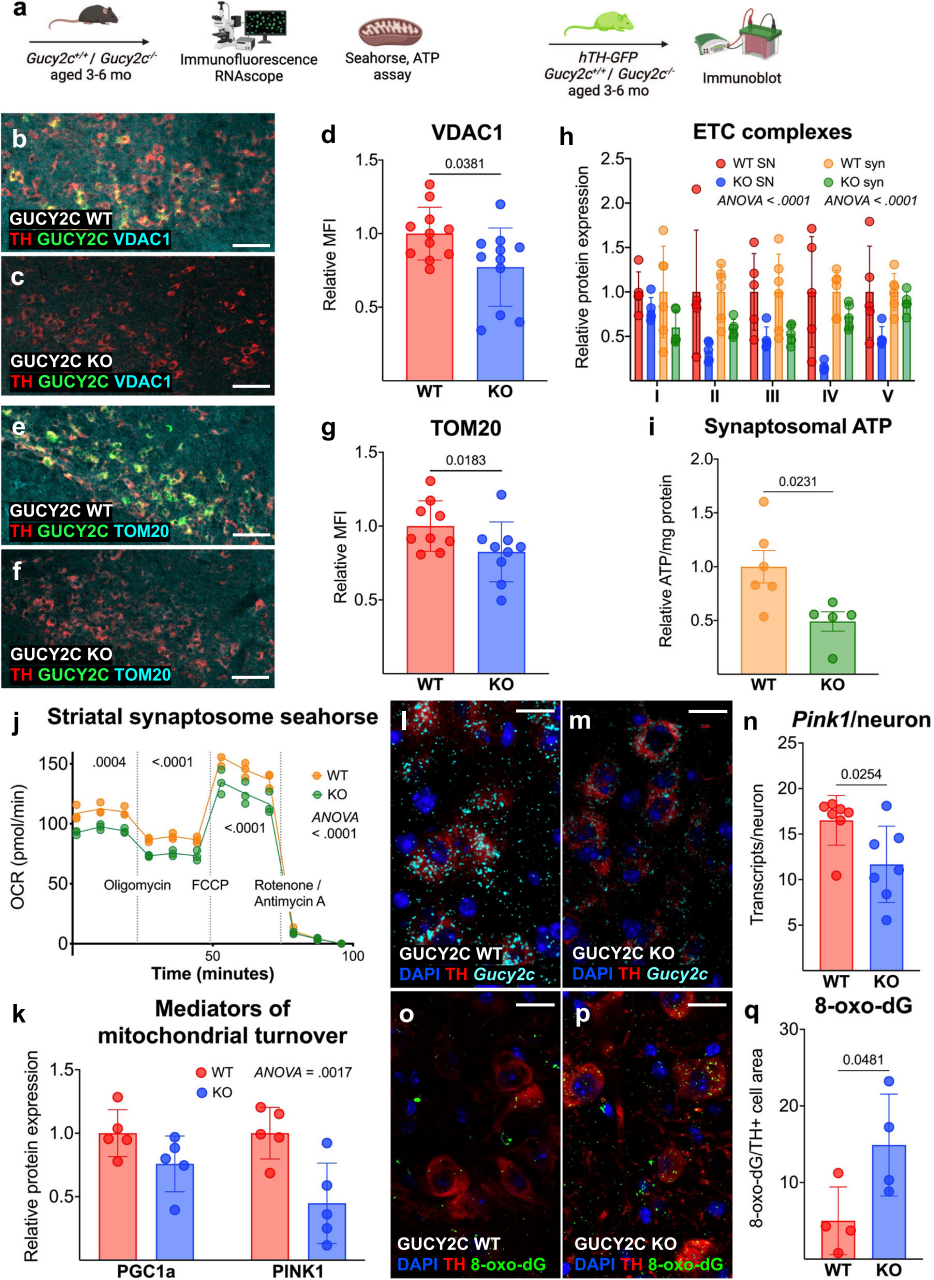
Loss of GUCY2C leads to mitochondrial dysfunction and oxidative stress within the nigrostriatal pathway. **a** Schematic of mice used for analyses. **b**–**g** Immunofluorescence of mouse midbrain reveals lower levels of **b**–**d** voltage-dependent anion-selective channel 1 (VDAC1) and **e**–**g** translocase of outer mitochondrial membrane 20 (TOM20) in *Gucy2c*^−/−^ (KO) substantia nigra pars compacta (SNpc) (*n* = 9–11). Scale bars represent 80 µM. **h** Protein isolated from tyrosine hydroxylase (TH)-GFP *Gucy2c*^+/+^ (WT) and *Gucy2c*^−/−^ SNpc and striatal synaptosomes reveal a lower level of electron transport chain (ETC) complex proteins in the *Gucy2c*^−/−^ nigrostriatal pathway (*n* = 5). **i** *Gucy2c*^−/−^ striatal synaptosomes produce lower levels of ATP at baseline (*n* = 5–6). **j** Seahorse analysis reveals lower oxygen consumption rates (OCR) in *Gucy2c*^−/−^ striatal synaptosomes (*n* = 3). **k** *Gucy2c*^−/−^ SNpc expresses lower protein levels of major drivers of mitochondrial turnover, peroxisome proliferator-activated receptor gamma coactivator 1-alpha (PGC1a) and PTEN-induced kinase (PINK1), quantified by immunoblot (*n* = 5). **l**–**n** *Gucy2c*^−/−^ mice express fewer *Pink1* mRNA transcripts within dopaminergic (DA) neurons quantified by RNAscope (*n* = 7). Scale bars represent 20 µM. **o**–**q** Immunofluorescence of 8-hydroxy-2- deoxyguanosine (8-oxo-dG) in *Gucy2c*^+/+^ and *Gucy2c*^−/−^ SNpc reveals greater levels of mitochondrial DNA (mtDNA) oxidation in *Gucy2c*^−/−^ (*n* = 4). Scale bars represent 20 µM. Statistics were calculated using two-tailed *t*-tests (**d**, **g**, **i**, **n**, **q**) or two-way ANOVA with a false discovery rate <0.05 (**h**, **j**, **k**). All immunoblot analyses were performed using microdissected TH-GFP reporter tissue. All error bars displayed represent the standard error of the mean (SEM).

We examined mitochondrial integrity and oxidative stress in *Gucy2c*^+/+^ and *Gucy2c*^−/−^ mice (Fig. 2a). Previously, we demonstrated that GUCY2C promotes mitochondrial function in the gut by maintaining mitochondrial protein content and oxygen consumption in intestinal epithelium^23^. In that context, loss of GUCY2C in the SNpc leads to lower levels of outer mitochondrial proteins voltage-dependent anion-selective channel 1 (VDAC1) and translocase of outer mitochondrial membrane 20 (TOM20)^24^ (Fig. 2b–g). Similarly, SNpc isolated from *Gucy2c*^−/−^ mice expressing a TH-GFP reporter express lower levels of mitochondrial ETC complexes compared to those from WT mice expressing the reporter (Fig. 2h and Supplemental Fig. 2a). SNpc DA neurons project to the dorsolateral striatum^25^, where DA is released from mitochondria-rich synapses^26,27^. Synaptosomes, positive for post-synaptic density 95 (PSD95) and synaptobrevin (synbrevin) isolated from this region (Supplemental Fig. 2b, c) in GUCY2C KO mice express lower levels of ETC proteins (Fig. 2h and Supplemental Fig. 2d–f), produce lower levels of adenosine triphosphate (ATP) (Fig. 2i), and have a reduced oxygen consumption rate (Fig. 2j). Furthermore, proteins that mediate mitochondrial turnover, including peroxisome proliferator-activated receptor gamma coactivator 1-alpha (PGC1a), a cGMP-responsive transcription factor that promotes mitochondrial biogenesis^28^, and PINK1, which mediates mitophagy^29^, are reduced in *Gucy2c*^−/−^ SNpc (Fig. 2k and Supplemental Fig. 2g). Within neurons, *Pink1* mRNA is trafficked to support local mitophagy, and *Pink1* transcripts have exceptionally short half-lives when unbound from damaged mitochondria^30,31^. This tightly controlled post-transcriptional regulation offers *Pink1* mRNA analysis as an additional readout of mitochondrial dynamics. In alignment with our protein data, *Pink1* mRNA transcript levels are significantly reduced in individual *Gucy2c*^−/−^ DA neurons (Fig. 2l–n). These findings are especially interesting, given that autosomal recessive mutations in the *Pink1* gene predispose individuals to hereditary PD^32^. GUCY2C KO mice also have more oxidized mitochondrial DNA (mtDNA) within DA neurons, indicative of a higher ROS burden (Fig. 2o–q)^33^.

### GUCY2C KO mice have an enhanced vulnerability to MPTP

**Fig. 3:**
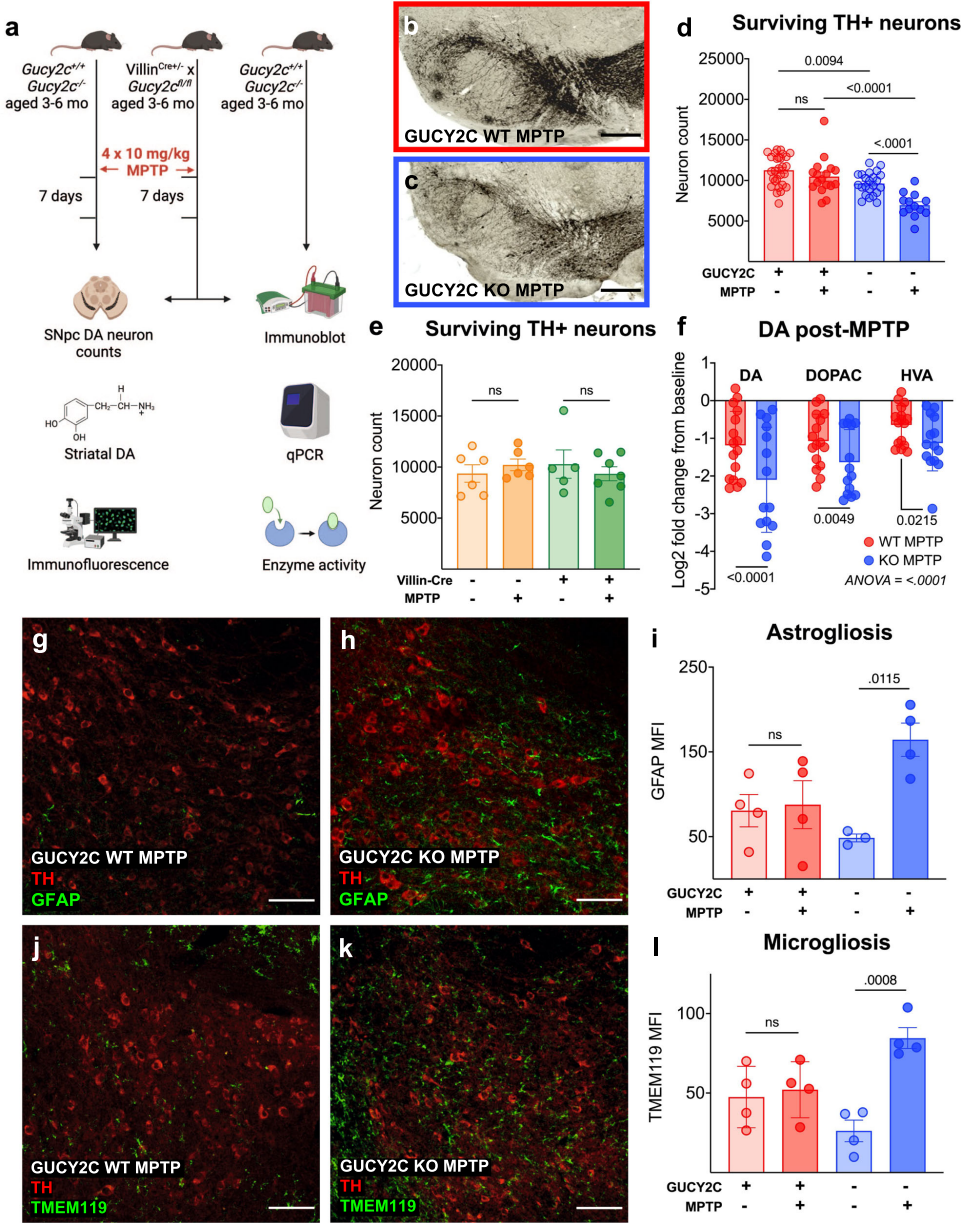
*Gucy2c*^*−/−*^ mice have an enhanced vulnerability to mitochondrial toxin MPTP. **a** Schematic of mice used for analyses. 1-methyl-4-phenyl-1,2,3,6-tetrahydropyridine (MPTP) mice received four intraperitoneal (IP) injections of 10 mg/kg and were sacrificed 7 days post-injection. Counting surviving tyrosine hydroxylase (TH)+ neurons in (**b**–**d**) IHC-stained substantia nigra pars compacta (SNpc) reveal that *Gucy2c*^−/−^ mice have fewer dopaminergic (DA) neurons at baseline and lose significantly more DA neurons post-MPTP (*n* = 13–28). Scale bars represent 200 µM. **e** Neuron loss is negligible post-MPTP in both Villin^cre+^ x *gucy2c*^fl/fl^ and Villin^cre−^ x *gucy2c*^fl/fl^ mice, indicating that loss of intestinal GUCY2C does not increase vulnerability to MPTP-induced DA neurodegeneration (*n* = 5–7). **f** HPLC reveals that *Gucy2c*^−/−^ mice lose significantly higher levels of DA, 3,4-dihydroxyphenylacetic acid (DOPAC), and homovanillic acid (HVA) upon MPTP injection. (*n* = 13–16). **g**–**l** Immunofluorescence analysis of MPTP SNpc demonstrates that levels of **g**–**i** astrogliosis (GFAP) and **j**–**l** microgliosis (TMEM119) are significantly greater in *Gucy2c*^−/−^ mice (*n* = 3–4). Scale bars represent 80 µM. Statistics were calculated using one-way ANOVA with a false discovery rate <0.05 (**d**, **e**, **i**, **l**), or by two-way ANOVA with a false discovery rate <0.05 (**f**). All error bars displayed represent the standard error of the mean (SEM).

Since mitochondrial dysfunction is a major driver of PD, we hypothesized that *Gucy2c*^−/−^ mice with mitochondrial deficits should be uniquely vulnerable to neurodegeneration induced by MPTP (Fig. 3a), a mitochondrial toxin that selectively targets DA neurons and induces cell death through oxidative stress^14,34^. As a standard dose of MPTP proved lethal to all *Gucy2c*^−/−^ mice (Supplemental Fig. 3a), we injected *Gucy2c*^+/+^ and *Gucy2c*^−/−^ with a subtoxic dose. At baseline, *Gucy2c*^−/−^ mice have fewer TH + DA neurons in the SNpc, and subtoxic doses of MPTP induce further loss of SNpc DA neurons in *Gucy2c*^−/−^, but not *Gucy2c*^+/+^, mice (Fig. 3b–d). This neurodegeneration is limited to the SNpc; subtoxic MPTP does not induce neurodegeneration in the *Gucy2c*^+^^/+^ or *Gucy2c*^−/−^ VTA (Supplemental Fig. 3b–f). Importantly, conditionally eliminating GUCY2C only in the intestine, but not in the brain (Supplemental Fig. 3g, h), did not amplify DA neuron vulnerability to subtoxic doses of MPTP (Fig. 3e), highlighting the role of SNpc GUCY2C in neuroprotection. Beyond the loss of DA neurons, subtoxic MPTP induces greater loss of striatal DA and its metabolites in *Gucy2c*^*−*^^/−^, as compared to in *Gucy2c*^+^^/+^, mice (Fig. 3f). Interestingly, *Gucy2c*^−/−^ mice have elevated levels of DA and DOPAC, but not HVA, at baseline compared to *Gucy2c*^+/+^ mice (Supplemental Fig. 3i–k), and DA metabolism is comparable between genotypes before and after exposure to MPTP (Supplemental Fig. 3l). Subtoxic MPTP also induces reactive astrogliosis and microgliosis in SNpc from *Gucy2c*^−/−^, but not *Gucy2c*^+/+^, mice (Fig. 3g–l). Increased toxicity of MPTP in *Gucy2c*^−/−^, compared to *Gucy2c*^+/+^, mice does not reflect a downregulation of VMAT2 or overexpression of DAT within DA neurons (Supplemental Fig. 3m), or an increase in the protein expression or activity of MAO-B, the enzyme which converts MPTP to the proximal toxin MPP+ (Supplemental Fig. 3n–p)^13,35,36^. The differential neurodegeneration between genotypes is not due to a more rapid response in *Gucy2c*^−/−^ mice; subtoxic MPTP does not induce DA neurodegeneration at either 7 or 14 days post-injection in *Gucy2c*^+/+^ mice (Supplemental Fig. 3q, r). Furthermore, phosphorylated alpha-synuclein (p-α-Syn), characteristic of PD and detectable in some chronic mouse models of neurodegeneration such as pre-formed fibril (PFF) injection^37^, is absent from DA neurons in both genotypes following subacute, subtoxic MPTP injections (Supplemental Fig. 3s–v).

### GUCY2C is overexpressed in pathology

**Fig. 4:**
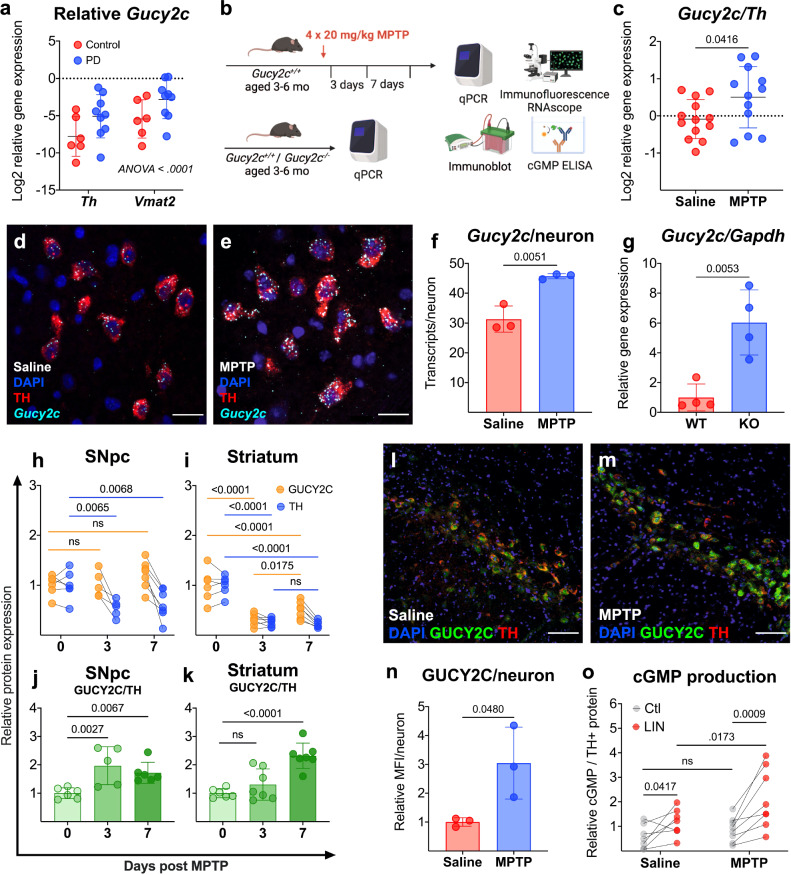
GUCY2C is overexpressed in pathology. **a** *Guanylyl cyclase C (Gucy2c)/tyrosine hydroxylase (Th)* and *Gucy2c/*v*esicular monoamine transporter 2 (Vmat2)* mRNA is elevated in PD patient substantia nigra pars compacta (SNpc) quantified from microarray analyses (*n* = 6–9). **b** Schematic of mice used for analyses. 1-methyl- 4-phenyl-1,2,3,6-tetrahydropyridine (MPTP) mice received 4 × 20 mg/kg of MPTP and were sacrificed 3 days (for RNA and protein analysis) or 7 days (for protein and ELISA analysis) post-injection. **c** *Gucy2c/Th* mRNA is elevated in SNpc isolated from MPTP mice, quantified by qPCR (*n* = 12–13). Scale bars represent 20 µM. **d**–**f** RNAscope and immunofluorescence of the SNpc of mice treated with **d** saline or **e** MPTP reveals **f** elevated *Gucy2c* mRNA transcripts in TH+ dopaminergic (DA) neurons following MPTP exposure (*n* = 3). **g** *Gucy2c*^−/−^ (KO) mice upregulate *Gucy2c* transcription, compared to *Gucy2c* ^+/+^ (WT) mice, quantified by qPCR (*n* = 4). Immunoblot analysis (**h**–**k**) reveals that TH is differentially reduced, compared to GUCY2C, in the nigrostriatal pathway following MPTP exposure (**h**, **i**), associated with an **j**, **k** elevated GUCY2C/TH ratio in the SNpc and striatum (*n* = 5–7). **l**–**n** Immunofluorescence analysis reveals that individual TH + DA neurons within the SNpc express significantly more GUCY2C protein 7 days post-MPTP injections (*n* = 3). Scale bars represent 80 µM. **o** Compared to SNpc isolated from saline-treated mice, ex vivo MPTP SNpc produces significantly more cGMP per TH+ protein upon GUCY2C agonist linaclotide (LIN), but not inactive peptide control, treatment (*n* = 7–8). This finding suggests that MPTP exposure induces a higher expression of GUCY2C on the surface of surviving DA neurons. Statistics were calculated using a two-way ANOVA with a post hoc false discovery rate <0.05 (**a**, **h**, **i**, **o**), a two-tailed *t*-test (**c**, **f**, **g**, **n**), or a one-way ANOVA with a post hoc false discovery rate <0.05 (**j**, **k**). All error bars displayed represent the standard error of the mean (SEM).

Publicly available transcriptomic data reveal that *Gucy2c* mRNA is overexpressed in DA neurons in PD patients (Fig. 4a) (GEO GSE42966). Similarly, *Gucy2c*^+/+^ mice treated with toxic doses of MPTP (Fig. 4b) overexpress *Gucy2c* mRNA in the SNpc (Fig. 4c). MPTP-induced upregulation in *Gucy2c* mRNA is likely caused by a compensatory increase of *Gucy2c* transcription in surviving DA neurons, rather than a selective survival advantage of neurons over-expressing *Gucy2c* (Fig. 4d–f). In contrast to the SNpc, *Gucy2c* mRNA expression is unchanged in the VTA post-MPTP injection (Supplemental Fig. 4a), consistent with the relative resilience of the VTA to DA neurodegeneration^38–41^. Overexpression of *Gucy2c* mRNA in PD patients and mice treated with MPTP mirrors our findings in SNpc isolated from *Gucy2c*^−/−^ mice (Fig. 4g), which were generated by inserting a neomycin cassette into exon 1 of the *Gucy2c* gene, allowing expression of *Gucy2c* mRNA, but not GUCY2C protein^42^. As these mice have dysfunctional mitochondria and elevated ROS levels at baseline (Fig. 2b–q), these data suggest that *Gucy2c* overexpression in the SNpc correlates with both chronic and acute oxidative stress and cellular damage. In WT mice, increased *Gucy2c* mRNA in the SNpc post-MPTP is associated with significantly higher expression of GUCY2C protein in the nigrostriatal pathway (Fig. 4h–n and Supplemental Fig. 4b). While GUCY2C/TH mRNA and protein levels significantly increase in the SNpc three days post-MPTP, increases in striatal GUCY2C/TH protein levels are only observable 7 days post-injection.

This delay may reflect the transit time of trafficking translated GUCY2C protein from the SNpc to the striatum^20^. Increased GUCY2C protein is expressed on the neuronal surface, and stimulating ex vivo samples with LIN, which specifically binds to the extracellular GUCY2C receptor domain, induces significantly greater production of cGMP in SNpc isolated from MPTP-treated mice (Fig. 4o).

### Cyclic GMP promotes antioxidant capacity in vitro and protects DA neurons from oxidative stress

**Fig. 5:**
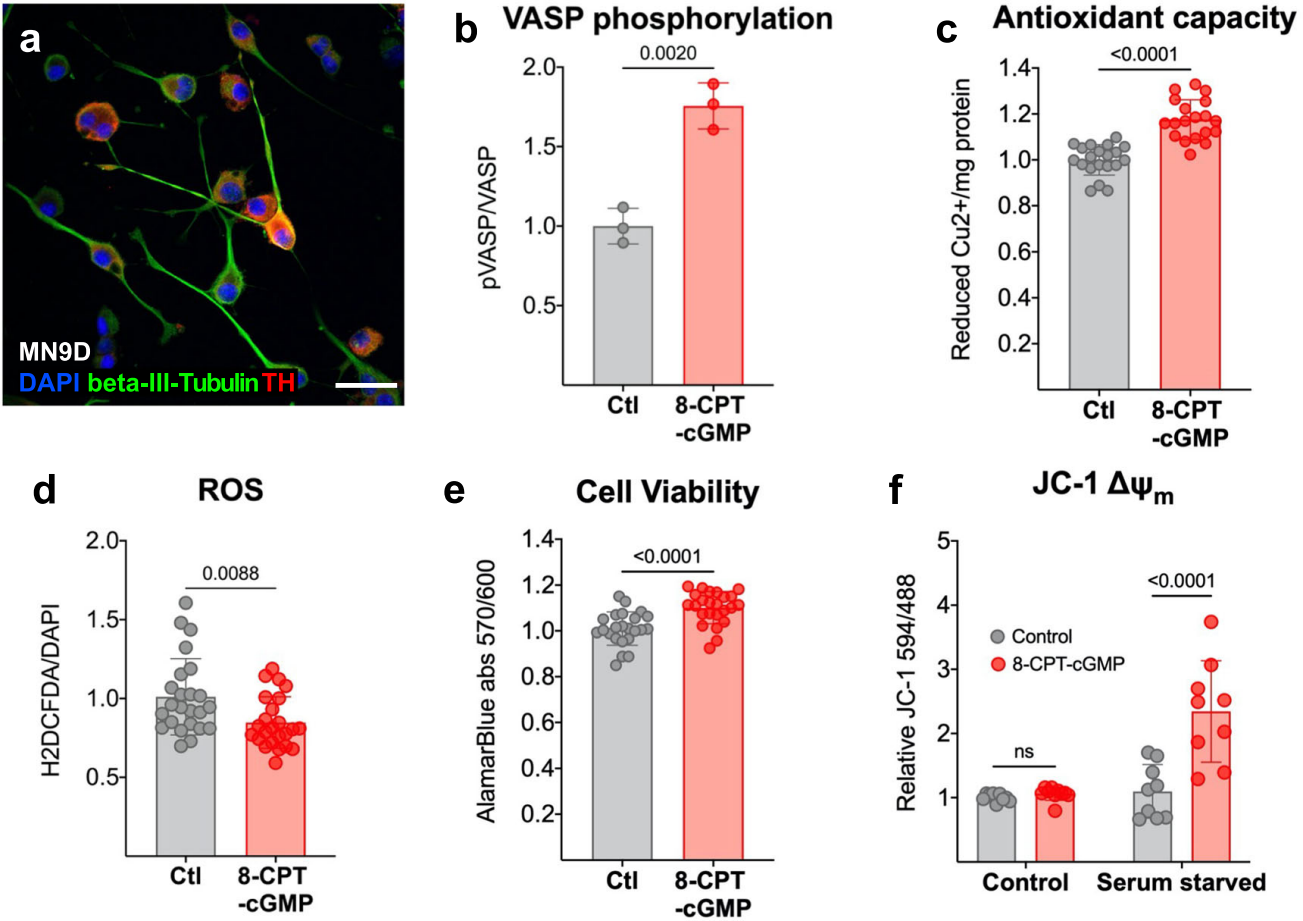
Cyclic GMP promotes antioxidant capacity in vitro and protects DA neurons from oxidative stress. **a** Mouse-derived MN9D neurons (scale bar represents 40 µM) accumulate **b** phosphorylated vasodilator-stimulated phosphoprotein at serine 239 (pVASP^ser239^) in response to cell- permeable cGMP analog 8-(4-chlorophenylthio)guanosine-3′, 5′-cyclic monophosphate (8-pCPT- cGMP) (*n* = 3) as quantified by immunoblot analysis. 8-pCPT-cGMP increases **c** antioxidant capacity (*n* = 24), which correlates with **d** reduced cellular reactive oxygen species (ROS) in 8- pCPT-cGMP-treated cultures, quantified by chloromethyl derivative 2ʹ,7ʹ- dichlorodihydrofluorescein diacetate (CM-H2DCFDA) staining (*n* = 12). **e** 8-pCPT-cGMP increases cell viability quantified by alamarBlue^TM^ reduction (*n* = 24). **f** Serum starvation reveals that 8-pCPT-cGMP mitigates the loss of mitochondrial membrane potential (ΔΨ_m_) in culture conditions that elevate oxidative stress (*n* = 9). For both conditions in (**f**), samples treated with 8- pCPT-cGMP are normalized to saline-treated (control) cells. Statistics were calculated using a two-tailed *t*-test (**b**–**e**) and a two-way ANOVA with a post hoc false discovery rate <0.05 (**f**). All error bars displayed represent the standard error of the mean (SEM).

The protective role of cGMP, the product of GUCY2C signaling, was explored in cultured MN9D mouse DA neurons^43^, which do not express detectable GUCY2C protein. Treating MN9D neurons (Fig. 5a) with the cell-permeable cGMP analog 8-(4-chlorophenylthio)guanosine- 3′, 5′- cyclic monophosphate (8-pCPT-cGMP) induces phosphorylation of vasodilator-stimulated phosphoprotein (VASP) at serine 239 (Fig. 5b and supplemental Fig. 5), confirming the capacity of these cells to respond to cGMP^44^. 8-pCPT-cGMP increases antioxidant capacity (Fig. 5c), reduces cellular ROS (Fig. 5d), and increases viability (Fig. 5e) of MN9D neurons. The protective effects of cGMP signaling are further pronounced upon exposure to oxidative stress. Under standard culture conditions, 8-pCPT-cGMP treatment of MN9D neurons has no impact on relative mitochondrial membrane potential (ΔΨ_m_; Fig. 5f), a marker of mitochondrial health^45^. However, 8-pCPT-cGMP significantly increases ΔΨ_m_ in neurons cultured in serum-starved media, a condition that induces oxidative stress^46^.

### Cyclic GMP reduces MPP^+^-induced oxidative stress, mitochondrial dysfunction, and cell death

**Fig. 6:**
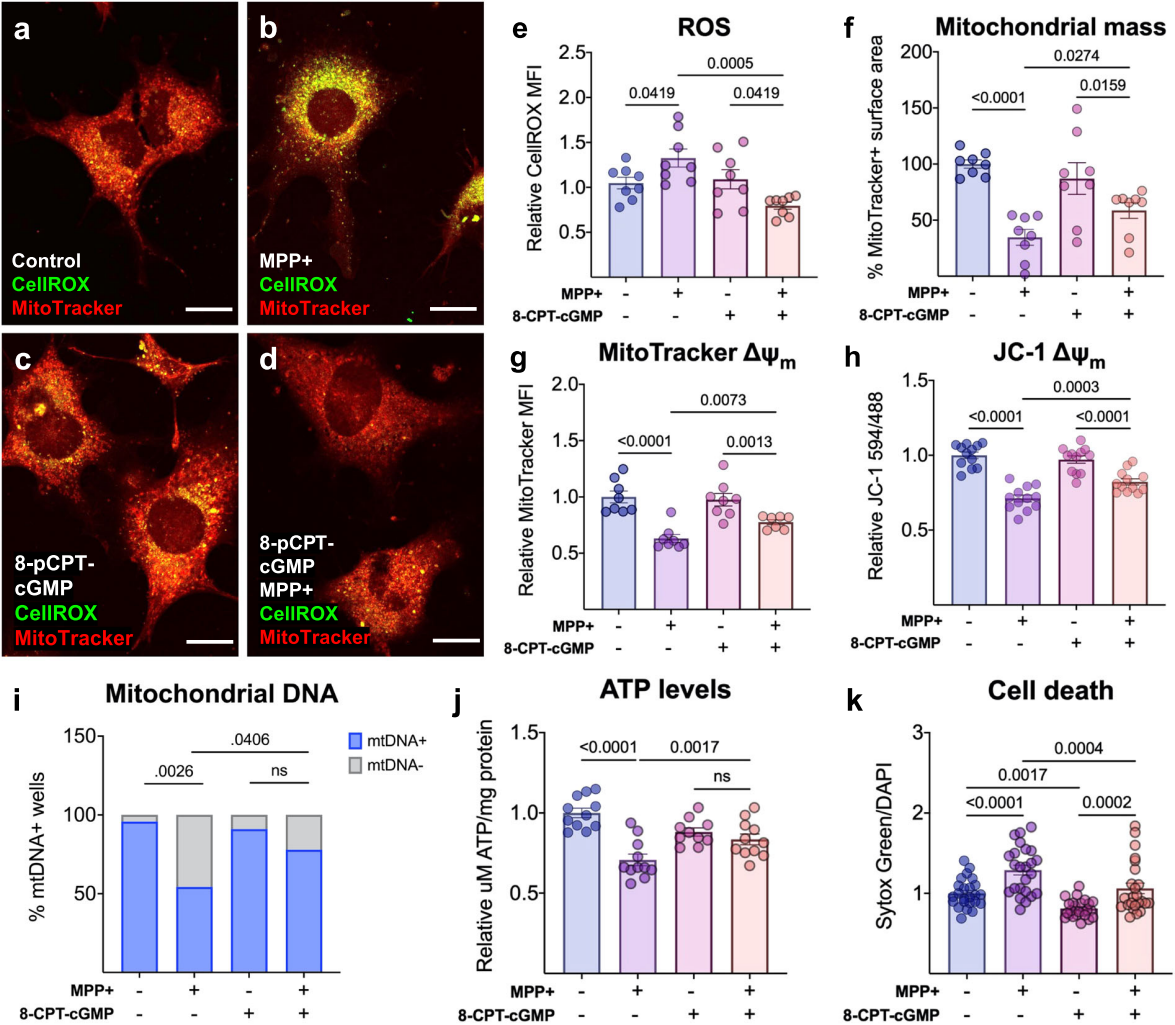
Cyclic GMP reduces MPP^+^-induced oxidative stress, mitochondrial dysfunction, and cell death. Cell-permeable cGMP analog 8-(4-chlorophenylthio)guanosine-3′, 5′-cyclic monophosphate (8- pCPT-cGMP) pretreatment rescues MN9Ds from 1-methyl-4-phenylpyridinium (MPP+)-induced **a**–**e** reactive oxygen species (ROS) accumulation as determined by CellROX fluorescence (*n* = 8, scale bars represent 20 µM) and mitigates MPP^+^-induced loss of **f** mitochondrial mass (*n* = 8) and ΔΨ_m_ (**g**, **h**), as determined by MitoTracker uptake (*n* = 8) and JC-1 dye fluorescence in live cells (*n* = 10–12). Also, 8-pCPT-cGMP reduces MPP^+^-induced loss of **i** mtDNA (*n* = 19–22), **j** ATP (*n* = 12), and **k** cell death quantified by Sytox green normalized to DAPI fluorescence (*n* = 22–24). Statistics were calculated using a one-way ANOVA with a post hoc false discovery rate <0.05 (**e**–**k**). All error bars displayed represent the standard error of the mean (SEM).

To further determine the ability of cGMP signaling to maintain mitochondrial integrity and promote cell survival during rising levels of ROS, we next investigated the potential of cGMP signaling to protect against MPP^+^-induced mitochondrial toxicity. Preconditioning with 8- pCPT-cGMP protects MN9D neurons against MPP^+^-induced ROS accumulation (Fig. 6a–e) and loss of mitochondrial mass (Fig. 6f), ΔΨ_m_ (Fig. 6g, h), mitochondrial DNA (Fig. 6i), and ATP (Fig. 6j). Critically, while 8-pCPT-cGMP alone promotes MN9D neuron viability, preconditioning also rescues these neurons from MPP^+^-induced cell death (Fig. 6k).

## Discussion

Here, we reveal a protective role for GUCY2C against DA neuron degeneration. Functional GUCY2C is expressed by DA neurons in the nigrostriatal pathway, where it maintains mitochondrial content and function while limiting ROS accumulation. GUCY2C protects against neurodegeneration in MPTP mice, and cGMP, the mediator of GUCY2C signaling, plays a parallel role in reducing oxidative stress, protecting mitochondria from injury, and decreasing cell death in DA neurons in vitro. GUCY2C is overexpressed in DA neurons in the SNpc of patients with PD and mice treated with MPTP. These observations suggest that stimulating GUCY2C may be effective in preventing nigrostriatal neurodegeneration with limited off-target effects. Additionally, GUCY2C overexpression in pathology indicates the potential of GUCY2C as a biomarker of early PD pathology.

Our findings align with established data linking impaired mitochondria and increased ROS levels with PD development and susceptibility to neurodegeneration in mouse models of Parkinsonism^15–18,47^. Post-mortem analysis reveals that PD patients have elevated ROS in the nigrostriatal pathway, and defects in ETC activity, mitochondrial biogenesis, mitophagy, and mitochondrial trafficking have been implicated in familial and idiopathic PD^15–18^. In vivo experiments have determined that compensating for these deficiencies by upregulating antioxidant activity or restoring mitochondrial integrity by enhancing mitochondrial biogenesis, mitophagy, or even direct mitochondrial infusion rescues mice from MPTP pathology^48–50^.

Further studies are necessary to define the protective mechanisms of GUCY2C signaling in DA neurons. Although a specific role for GUCY2C in protecting against neurodegeneration is an emerging frontier, cGMP signaling protects mitochondria, reduces ROS, and preserves survival across different cell types. In some cells, cGMP induces mitochondrial biogenesis by upregulating PGC1a^51,52^, a master transcription factor crucial to de novo mitochondria synthesis. Empagliflozin, a sodium-glucose transport inhibitor used to treat diabetes, recently was demonstrated to play a separate role in reducing oxidative stress and cell death by cGMP signaling in a mouse model of diabetic cardiomyopathy^53^. Moreover, cGMP upregulates antioxidant genes in colon, lung, and heart^54–56^. Also, cGMP promotes homeostatic autophagy, a process dysregulated in PD^57,58^, and at low levels, cGMP blocks apoptosis in cultured neuronal and neuroblastoma cells^59,60^. However promising these in vitro results may be for PD research, in vivo experiments investigating the role of cGMP in animal models of DA neurodegeneration have yielded conflicting results^61,62^. As previous experiments were performed using phosphodiesterase (PDE) inhibitors, which prevent degradation of cyclic nucleotides across a broad range of cells and pathways within the CNS, our study of a guanylyl cyclase selectively expressed by DA neurons reveals the potential to raise cGMP specifically within the nigrostriatal pathway in vivo.

To explain impaired mitochondria, elevated ROS levels, and susceptibility to neurodegeneration in *Gucy2c*^−/−^ mice, we have considered multiple potential mechanisms that may cause ROS overproduction or limit ROS scavenging in *Gucy2c*^−/−^ DA neurons. One possible cause of ROS overproduction begins with impaired mitochondrial dynamics. *Gucy2c*^−/−^ SNpc express lower levels of PGC1a, a driver of mitochondrial biogenesis, and PINK1, a mediator of mitophagy and an indirect PGC1a activator that, when mutated, is associated with PD^63,64^. Combined with lower levels of mitochondrial proteins, oxygen consumption, and ATP in the nigrostriatal pathway, our findings suggest that *Gucy2c*^−/−^ mice may have defective mitochondrial production and turnover within DA neurons. This relatively limited and inefficient pool of mitochondria may underlie the observed increase of oxidative stress within the *Gucy2c*^−/−^ SNpc, as mitochondria are the primary source of cellular ROS^65,66^. During homeostasis, ROS generated by oxidative phosphorylation, including superoxide, hydrogen peroxide (H_2_O_2_), and hydroxyl radical, are scavenged by antioxidants, such as glutathione, SOD2, and catalase^65^. However, these endogenous defenses are overwhelmed when ROS production is significantly elevated in pathological conditions, such as when the ETC is impaired^67^, as observed in *Gucy2c*^−/−^ mice.

Alternatively, the inciting source of potential ROS accumulation in *Gucy2c*^−/−^ DA neurons may precede mitochondrial dysfunction. In contrast to a previous study yielding no differences in striatal DA levels in *Gucy2c*^−/−^ mice^68^, we detected a modest, yet significant, increase in DA in the *Gucy2c*^−/−^ striatum. This finding is particularly interesting as *Gucy2c*^−/−^ have fewer DA neurons within the SNpc, suggesting an overproduction of DA per neuron. DA neurons are particularly vulnerable to oxidative stress in part because DA production and degradation yield substrates for autoxidation^69^, which is typically offset by endogenous mechanisms. A recent study reveals that MAOs tethered to mitochondria in DA neurons redirect H_2_O_2_, a by-product of DA deamination to DOPAC, into complex IV of the ETC, thus limiting the ROS burden of DA metabolism while generating ATP^70^. However, as *Gucy2c*^−/−^ mice produce more DA, but express lower levels of ETC proteins, they may simultaneously generate more ROS and lack the ability to reduce the resulting oxidative stress. As elevated oxidative stress induces DNA damage, peroxidation of the lipid membrane, and protein oxidation, the vicious cycle of ROS production and mitochondrial damage may be amplified in *Gucy2c*^−/−^ mice^71–73^, and may explain the mitochondrial dysfunction and reduced SNpc DA neuron population in aged *Gucy2c*^−/−^ mice as compared to age-matched controls. Further studies quantifying DA neuron ROS and comparing the number of SNpc DA neurons in *Gucy2c*^+/+^ and *Gucy2c*^−/−^ from embryogenesis to adulthood are required to determine whether the reduced population of DA neurons within the aged *Gucy2c*^−/−^ SNpc is due to increased accumulation of age-related cellular damage or a defect in early neuronal development.

Still another possible explanation of elevated oxidative stress and dysfunctional mitochondria is impaired ROS scavenging in *Gucy2c*^−/−^, which is supported by our in vitro findings. We and others have demonstrated that cGMP signaling promotes antioxidant function across a wide range of cell types, including cultured DA neurons^54–56^. However, unlike our findings in vivo, our in vitro studies did not reveal a role for cGMP in promoting mitochondrial integrity in the absence of acute oxidative stress. This discrepancy may be due to the relatively short incubation time of cultured DA neurons with cGMP in contrast to the long-term impact of mice aging with competent GUCY2C expressed by SNpc DA neurons. Regardless, the precise role of GUCY2C in preventing ROS accumulation and mitochondrial dysfunction in DA neurons remains undefined and is a topic for future investigation. Comparing specific antioxidant enzyme activity between genotypes, treating *Gucy2c*^+/+^ and *Gucy2c*^−/−^ mice exposed to MPTP with antioxidants, and performing long-term in vitro characterizations of mitochondria within DA neurons supplemented with cGMP may help to determine the role of GUCY2C on ROS overproduction and scavenging.

Further complicating the directionality between GUCY2C, mitochondrial integrity, ROS production, and ROS scavenging is the apparent feedback loop between oxidative stress and cGMP signaling that is suggested from our data and may be conserved across species. Recent work exploring the relationship of ROS and cGMP in *C. elegans* revealed that mutated mitochondrial ETC complexes I or III or exposure to paraquat, a lipid membrane oxidation agent, increases cGMP accumulation and is prevented by pretreatment with antioxidants^74^. This finding parallels our mouse data; MPTP upregulates *Gucy2c* mRNA in the SNpc, but not in the VTA, which is comparitively resilient to mitochondrial toxins and has lower levels of oxidative stress at baseline^38–40^. This observation supports the hypothesis that *Gucy2c* transcription and potential for elevated cGMP production is closely regulated by ROS sensors. The dynamism of GUCY2C-cGMP signaling in pathology is not limited to the SNpc, although there are variations across organ systems that may reflect intrinsic differences in cell type or the source of cellular stress. Like in the SNpc, mice fed a high-fat diet, which induces neuroinflammation in the hypothalamus, overexpress hypothalamic *Gucy2c* mRNA and protein and undergo increased cGMP-driven VASP phosphorylation upon ligand treatment^75^. Similar to changes in GUCY2C+ pathological brain regions, GUCY2C is overexpressed in over 95% of colorectal tumors, as well as in a subset of pancreatic, gastric, and esophageal tumors^76^, and is currently under investigation as a potential therapeutic target for treating GI cancers^77^. While interesting to note, alterations in intestinal and neural GUCY2C expression and signaling in pathology remain an emerging field. Further studies are required to characterize GUCY2C expression and activity in the context of a wide range of stressors and identify the mechanisms driving these changes.

Although our data suggest that the GUCY2C is neuroprotective against MPTP, endogenous overexpression of the protein in pathology is insufficient to prevent neurodegeneration. One possible explanation is that the amount of GUCY2C expressed may vary between DA neurons. Neurons that express higher levels of GUCY2C may be more resilient to oxidative stress, leading to a selective loss of neurons that express low levels of the receptor and subsequent enrichment of DA neurons that express higher GUCY2C levels in pathology. Another potential explanation is that there is a maximum overexpression and activity level of GUCY2C within neurons, which may only compensate for a modestly elevated burden of oxidative stress. While overexpression likely offers a degree of protection, after cells reach a tipping point of ROS, endogenous mechanisms such as antioxidant pathways and increased cGMP signaling may be overwhelmed. Finally, while GUCY2C is overexpressed on DA neurons in pathology, endogenous SNpc GUCY2C ligands remain unknown. Further studies are necessary to identify SNpc GUCY2C agonists, characterize their availability in pathology, and quantify ligand-induced GUCY2C signaling in physiological and pathological conditions. These data will help determine whether there is a gap in receptor expression and ligand availability, offering insight into the utility of ligand-replacement therapy to boost the activity of overexpressed SNpc GUCY2C and stimulate protective pathways to guard against DA neurodegeneration.

Untangling the relationship between oxidative stress, cGMP signaling, and GUCY2C expression holds critical implications for neurodegenerative research. Our study has identified SNpc *Gucy2c* as a potential transcriptional target of oxidative stress, which may offer overexpressed GUCY2C as an indicator of cellular damage and susceptibility to degeneration in PD. Currently, PD is diagnosed when patients lose 50–90% of the DA neurons within the SNpc and experience subsequent motor deficits^78^. Although DAT single-photon emission computed tomography (DAT SPECT) is a highly effective FDA-approved technique to distinguish PD from other pathologies that induce essential tremor, there are currently no biomarkers that can identify early pathology prior to profound neurodegeneration^5,48^. Our data reveal GUCY2C as a ROS-responsive surface receptor expressed on 98% of TH+ SNpc neurons that undergoes significant upregulation during pathology, although further studies are needed to track the relationship between chronic DA neuron loss and GUCY2C upregulation in surviving neurons. Furthermore, we have demonstrated the ability of linaclotide to specifically bind GUCY2C in ex vivo SNpc samples. These findings suggest that elevated GUCY2C may be detectable upon intravenous or intrathecal administration of radiolabeled ligands to reflect rising levels of oxidative stress in the early stages of neurodegeneration. Importantly, gamma camera imaging captures GUCY2C in patient-derived colorectal tumor xenografts in mice intravenously administered radiolabeled ligand^79^, demonstrating the utility of this approach for quantifying GUCY2C in the intestine. Further research is necessary to assess the ability of intravenous radiolabeled GUCY2C agonists to cross the blood-brain barrier to determine the most effective route of administration. Ultimately, clarifying the potential of administered ligands to bind SNpc GUCY2C may provide the foundation for multiple therapeutic applications. These include measuring increased GUCY2C expression levels with radiolabeled ligands to identify susceptibility to DA neurodegeneration and targeting SNpc GUCY2C with unlabeled ligands to stimulate protective cGMP signaling specifically within DA neurons.

Many open questions remain for future investigation. Although our data suggests the protective role of GUCY2C-cGMP signaling in the context of neurodegeneration, the endogenous ligand that stimulates SNpc GUCY2C remains undefined. However, data from our group and others suggest a potential gut–brain signaling axis between intestinal hormones and neural GUCY2C. Dysfunctional intestinal hormone signaling is an emerging field of study in the context of neurodegeneration. PD patients produce lower levels of circulating ghrelin and glucagon-like peptide-1 (GLP-1), gastrointestinal (GI) hormones best known for their role in mediating satiety through hypothalamic signaling^80^. Recent experiments have revealed that ghrelin and GLP-1 also have neuroprotective roles, such as reducing neuroinflammation and mitigating DA neuron cell loss in MPTP mice^81–84^. Similarly, we have demonstrated a gut–brain endocrine axis between uroguanylin, a hormone produced and released postprandially by the small intestine that can cross the blood-brain barrier, and hypothalamic GUCY2C in regulating satiety^75,85^. These findings suggest that intestinal uroguanylin may also serve as an endogenous ligand for GUCY2C expressed in the nigrostriatal pathway. Clinical studies comparing uroguanylin levels in fasted PD and healthy patients, as well as ligand depletion and uroguanylin overexpression experiments in MPTP mice, are critical to determine whether nigrostriatal GUCY2C is a component of a neuroprotective gut–brain axis. Alternatively, SNpc GUCY2C may respond to a currently unknown neural ligand. Immunoprecipitation and purification of SNpc GUCY2C, combined with mass spectrometry and confirmatory labeled ligand binding assays, may help to identify neural regulators of SNpc GUCY2C signaling. Finally, ligand-independent signaling may underlie the protective effects of GUCY2C in DA neurons. ELISA data reveals that despite increased GUCY2C protein in MPTP-treated DA neurons, cGMP levels are only elevated upon ligand stimulation. While this finding suggests that ligand-independent GUCY2C signaling is unlikely, mutating neural GUCY2C receptor sites in mice to prevent ligand binding and determining the resulting vulnerability to MPTP would more effectively demonstrate the role of ligand-dependent vs independent signaling in GUCY2C-mediated neuroprotection.

As multiple epidemiological and experimental studies have implicated altered GI function and a disrupted gut–brain signaling axis in PD pathology, future studies characterizing the impact of GUCY2C on neurodegeneration should not be limited to the role of GUCY2C in the brain. GI symptoms such as constipation and dysphagia frequently present in PD patients decades prior to motor deficits^86,87^, and α-Syn clusters spread from the intestine to the brain via the vagus nerve, propagating Lewy body formation throughout the CNS^88–90^. PD patients also have disrupted microbiomes, which may hold implications for neuroinflammation in pathology^91,92^. Interestingly, the therapeutic potential of fecal transplants to ameliorate motor symptoms in PD patients is currently being explored in a clinical trial (ClinicalTrials.gov identifier NCT03808389). While the loss of GUCY2C in mouse intestine does not increase vulnerability to subacute MPTP, intestinal GUCY2C may play a larger role in alternative models and mechanisms of DA neuron degeneration, such as migration of Lewy bodies from the GI tract to the brain and regulation of gut microbiota. Determining the susceptibility of global *Gucy2c*^−/−^ vs conditional knockout mice lacking GUCY2C in the intestine or DA neurons in other mouse models, such as PFF injection into the duodenum, may clarify neural and intestinal roles of GUCY2C in mitigating pathology.

## Methods

### Mice

Mice for these studies were bred, maintained, genotyped, and functionally characterized in the animal care facility at Thomas Jefferson University, and all protocols employed were pre-approved by the Institutional Animal Care and Use Committee. *Gucy2c*^−/−^ mice on a C57Bl6/J background are maintained within our colony^20,42,93^. *Gucy2c*^*fl*/*fl*^ mice were developed in conjunction with the CRISPR-Cas9 Mouse Targeting Core at the University of Pennsylvania (Philadelphia, PA) (RRID:SCR_022378). *hTH-GFP* mice were generated as described and maintained on a C57Bl6/J background^94^. *hTH-GFP* mice were crossed with *Gucy2c*^−/−^ mice to generate GUCY2C WT and KO reporter littermates. Villin^cre^ mice (Stock: 021504) were purchased from the Jackson Laboratory. Mice were raised with 12 h light and dark cycles and were used from age 12–24 weeks. All mice were compared to littermate controls or bred as F2 crosses of *Gucy2c*^+^^/+^ and *Gucy2c*^−/−^ from heterozygote parents.

### Brain immunofluorescence

Following perfusion of mice with ice-cold PBS and subsequent 4% paraformaldehyde, brains were extracted and incubated in 4% PFA at 4 °C for 24–48 h, and then cryoprotected by incubation in 30% sucrose in PBS at 4 °C until brains sank (24–72 h). Brains were frozen in OCT medium by submerging in dry ice-cooled methanol and stored at −80 °C until sectioning. Forty-micron sections were cut using a cryostat, and then stored floating in cryoprotectant until staining.

PFF-injected mice (described in greater detail below) underwent identical perfusion as described above. PFF brains were postfixed overnight, then dehydrated through graded ethanol, defatted in xylene, and embedded in the coronal plane in paraffin (ParaplastXtra; Thermo Fisher, Waltham, MA). Serial 10-μm sections (five sections per slide) were cut and mounted onto Superfrost™ Plus Microscope Slides (Thermo Fisher).

Each wash step below represents three consecutive 5-min incubations in PBS with 0.1% tween- 20 (PBST). Epitope retrieval was performed on floating and mounted sections by incubating in pH 9 retrieval solution (Agilent Technologies, Santa Clara, CA) for 20 min at 80 °C. Samples were blocked for 1 h in blocking buffer (10% milk (w/v) in PBS with 0.3% Triton-X), and then incubated, shaking in 1° antibody solution (diluted in blocking buffer) overnight at 4 °C. After a wash step, samples were incubated, shaking in blocking buffer with 2° antibody and nuclear counterstain DAPI for 60 min at room temperature. PBS was substituted with TBS for staining phosphorylated proteins. For all stains except GUCY2C, a fluorophore-conjugated 2° antibody was used. To stain GUCY2C, a peroxidase-conjugated 2° antibody was used, followed by tyramide-amplification: samples were washed and then incubated in tyramide-FITC, at a final concentration of 100 μg/mL in PBS with 0.003% H2O2 for 10 min (Hopman et al. 1998).

Following a final series of washes, floating samples were mounted onto Superfrost™ Plus Microscope Slides, and slides were coverslipped using Prolong Diamond antifade mounting media (Thermo Fisher). Antibody IDs and concentrations are listed in supplemental table 1. Confocal images were taken of 10 different sections from each mouse for each region. Cells were quantified from five different optical sections (at least 6-μm apart) for each section. Mean fluorescent intensity was calculated in ImageJ software.

### RNAscope

Mice were sacrificed 3 days post-MPTP injection. Following perfusion of mice with ice-cold PBS, brains were extracted, frozen in OCT medium by submerging in dry ice-cooled methanol, and stored at −80 °C until sectioning. Ten-micron sections were cut using a cryostat onto Superfrost™ Plus Microscope Slides (Fisher Scientific, 12-550-15) and stored at −80 °C until use. RNAscope stains used the reagents and staining protocol provided by Advanced Cell Diagnostics (ACD) USA (Newark, CA) (ACD, 323110). Each wash step below represents two consecutive 2-min incubations in ACD wash buffer. Briefly, slides were drop-fixed in ice-cold 4% PFA for 15 min, followed by 5 min sequential dehydration steps in ice-cold 50, 70, and 100% ethanol.

Sections were blocked with 3% hydrogen peroxide for 10 min, followed by two wash steps and incubation with RNAscope probes (Supplemental Table 1) for 2 h at 40 °C. Sections were then washed and incubated with AMP1 for 30 min, AMP2 for 30 min, and AMP3 for 15 min at 40 °C with wash steps after each incubation. Depending on the RNAscope probe channel, sections were then incubated with C1, C2, or C3 for 15 min at 40 °C, washed, and incubated with opal reagent 570 or 690 (Akoya Biosciences, Marlborough, MA) for 30 min at 40 °C. Sections were washed and incubated in provided HRP blocking for 15 min at 40 °C to complete the RNAscope stain. To combine RNAscope with immunofluorescence, sections were then blocked and counterstained with primary and secondary antibodies, following the immunofluorescence protocol detailed above (antibody concentrations for combined RNAscope and immunofluorescence are provided in Supplemental Table 1). DAPI was mixed into secondary antibodies to counterstain nuclei. Slides were coverslipped using Prolong Diamond antifade mounting media (Thermo Fisher). Confocal images were taken of eight different 10-μm sections from each mouse for each region. Cells were quantified from five different optical sections (at least 6-μm apart) for each 10-μM-thick section. Individual mRNA transcripts were analyzed in ImageJ software. The script for quantification is available upon request.

### Pretreatment of sections for 8-oxo-dG immunofluorescence

To detect 8-oxo-dG in mtDNA, free-floating sections were incubated in 10 mM Tris-HCl (pH 7.5), 15 mM NaCl containing DNase-free Rnase (5 mg/ml of heat-inactivated Rnase A, Sigma, Saint Louis, MO) for 60 min at 37 °C, prior to incubation with primary antibody. As a negative control, cellular DNA was eliminated by incubating sections in 50 mM Tris-HCl (pH 7.5), 0.1 mM MgCl_2_ containing Rnase-free Dnase I (1000 U/ml, Sigma) for 60 min at 37 °C, following incubation with Dnase-free Rnase. Antibody information is provided in Supplemental Table 1.

### Cyclic GMP ELISA

Midbrains were isolated from *Gucy2c*^+/+^ and *Gucy2c*^−/−^ mice on ice and minced in Neurobasal media (Thermo Fisher) supplemented with 1X N-2, B-27, and GlutaMAX (Thermo Fisher).

Minced midbrains were divided evenly into a 24-well plate containing 150 uL of supplemented Neurobasal media with 3-isobutyl-1-methylxanthine (IBMX); 1 mM and incubated for 20 min at 37 °C. About 150 uL of supplemented media containing 1 mM IBMX and either 2 µM linaclotide (Ironwood Pharmaceuticals, Cambridge, MA) or 2 µM TJU (inactive peptide control) for an additional 30 min at 37 °C. Subsequently, the protein was purified (detailed below) and analyzed for cGMP using a competitive cGMP ELISA kit according to manufacturer instructions (Cayman Chemical Company, Ann Arbor, MI).

### Protein isolation

SNpc from *hTH-GFP* x *Gucy2c*^+/+^ and *hTH-GFP* x *Gucy2c*^−/−^ was isolated under a fluorescent microscope. Tissue was immediately placed into ice-cold M-PER (Thermo Fisher) supplemented with protease and phosphatase inhibitors (Roche) and homogenized using a 1 mL insulin syringe (BD Biosciences). Striatal synaptosomes were isolated by homogenizing dorsolateral striata in Syn-PER (Thermo Fisher) supplemented with protease and phosphatase inhibitors (Roche) and centrifugation according to manufacturer instructions. Protein was quantified using the BCA assay (Thermo Fisher).

### Immunoblot analysis

Lysates were analyzed by SDS-PAGE (NuPAGE 4-to-12% bis-Tris gel; Novex Life Technologies) and electrophoretically transferred to a nitrocellulose membrane (Novex Life Technologies). Immunoblot membranes were cut at multiple molecular weights to allow for multiple analytes to be probed within individual experiments. The membrane was blocked with 5% bovine serum albumin (BSA) in PBST (1× PBS and 1% Tween 20) and probed overnight at 4 °C with primary antibodies detailed in supplemental table 1. The following day, membranes were washed three times with PBST and incubated with goat anti-mouse horseradish peroxidase (HRP)-conjugated and goat anti-rabbit HRP-conjugated secondary antibodies (1:10,000; Jackson ImmunoResearch) in 5% BSA in PBST for 1 h at room temperature. Blots were developed in SuperSignal West Dura, or Femto-enhanced chemiluminescence (ECL) substrate (Thermo Scientific). Relative intensity was quantified by densitometry using ImageJ and normalized to the intensity of GAPDH or HSP90. Housekeeping probes were chosen to minimize interference with proteins of interest, after confirming that *Gucy2c*^−/−^ expresses comparable levels of HSP90 as

*Gucy2c*^+/+^ (supplemental Fig. 2e, f). All blots were processed in parallel and derived from the same experiments.

### Synaptosome respirometry

For monitoring respiration, synaptosomes were resuspended in Seahorse medium (Agilent Technologies) (phenol-free DMEM pH 7.4, supplemented with 2 mM glutamine, 10 mM glucose, 1 mM pyruvate). Five-hundred µL of Seahorse medium containing 80 µg of synaptosomal protein per well was aliquoted into a 24-well cell culture microplate (Agilent Technologies) pre-coated with a 1:15,000 diluted polyethyleneimine (PEI). The plate was centrifuged at 3000 × *g* for 1 h at 4 °C and equilibrated to 37 °C prior to the experiment. The cell culture microplate was incubated and loaded into the Seahorse XF24 extracellular flux analyzer following the manufacturer’s instructions. All experiments were performed at 37 °C. Reagents were added at appropriate dilutions in the Seahorse medium (volumes and concentrations of Seahorse inhibitors provided in Supplemental Table 2). Three biological replicates and four technical replicates were run per plate.

### ATP assay

Synaptosomal and MN9D protein content was quantified prior to TCA deproteinization (Abcam, 204708). Ten μL of the deproteinized sample was incubated with fluorescent ATP assay kit reagents (Abcam 83355) in a glass-bottom black 96-well plate (Nalge Nunc International Corporation, Rochester, NY). Fluorescent ATP content was measured using a microplate reader at a 535/587 excitation/emission wavelength. Background levels of glycerol-3-phosphate were subtracted from each sample according to manufacturer instructions.

### Subtoxic MPTP

In experiments measuring GUCY2C levels following MPTP injections, *Gucy2c*^+/+^ mice received four intraperitoneal (IP) injections of vehicle or MPTP at 20 mg/kg (80 mg/kg total; free base in PBS, Sigma). As this dose is toxic for *Gucy2c*^−/−^ mice (Supplemental Fig. 3a), experiments comparing *Gucy2c*^+/+^ vs Gucy2c^−/−^ response to MPTP used four intraperitoneal (IP) injections of vehicle or MPTP at 10 mg/kg (40 mg/kg total; referred to as subtoxic MPTP). Seven days after the last injection, all mice were heavily anesthetized with avertin prior to perfusion.

### Preparation of α-Syn PFFs and quality control

Recombinant human α-Syn expressed in *E. coli* was obtained from Proteos (Kalamazoo, MI). The monomeric form of α-Syn was centrifuged at 4 °C for 10 mins at 15,000 × *g*. After centrifugation, the supernatant was removed, and its protein concentration was determined using a Nanodrop Model 2000 spectrophotometer (Fisher Scientific). For each sample, the monomer was diluted to a final concentration of 5 mg/ml in ~100 mM NaCl, ~7.5 mM Tris, and ~10 mM phosphate and adjusted to pH 7.4. A 100 μg aliquot was placed into an orbital shaker at 37 °C for 7 days at 1000 RPM to induce fibrillization.

### Preparation of surgical material

PFFs or monomeric α-Syn were diluted to 2 μg/μL in sterile dPBS. These samples were then sonicated using a microtip sonicator at power level 2 for 0.5 sd ON/0.5 s OFF pulses, with pausing every 10 s to prevent excess heat and frothing. Following sonication, successful disruption of the fibrils was confirmed using transmission electron microscopy, with the average fibril being ~50 nm in length. Monomeric protein was centrifuged at 15,000 × *g* at 4 °C before surgery, with the supernatant retained for use in surgical injections.

### Stereotaxic injection of PFFs and monomeric α-Syn into mouse brain

Mice were anesthetized using a 3% mixture of isoflurane/oxygen. Following exposure of the skull, a small hole was drilled using a robotic stereotaxic instrument (Neurostar, Tubingen, Germany) at locations above the striatum or hippocampus. Two μL of diluted monomers or PFFs (2 μg/μL) into either the dorsal lateral striatum (AP 0.86, DV -2.5, ML 1.8) or rostral hippocampus (AP -2.18, DV -1.25, ML 1.75) using a 10 μL Hamilton microsyringe at a constant rate of 0.4 μL per min. The syringe was left in place for 5 min after which it was slowly removed. The scalp was then closed using proline suture, and the wound was treated with topical lidocaine. The brain stained for phosphorylated α-Syn in supplemental Fig. 3t was isolated 60 days post-injection (dpi).

### TH Immunohistochemistry

Every fifth 40 μM floating section of mouse midbrains was prepared and stained with primary antibody overnight as described above. Next, the sections were incubated with an avidin–biotin–horseradish peroxidase complex (Vector Laboratories) according to the manufacturer’s instructions. The sections were stained with a DAB kit (Vector Laboratories) before mounting onto Superfrost™ Plus Microscope Slides and coverslipping using Diamond ProLong mounting media.

### TH+ neuron counts

TH+ neurons were visualized using a 100×, 1.3 numerical aperture objective (Olympus, Center Valley, PA) on a BX51 microscope (Olympus) with a MAC5000 motorized XYZ axis computer-controlled stage and a CX9000 CCD video camera (MicroBrightField). Neurons were counted using a fractionator-sampling design in morphometry and a design-based stereology software package, StereoInvestigator, (version 7.0; MicroBrightField, Colchester, VT, USA). Briefly, at least 8 sections of SNpc were traced per mouse at 4x magnification, and this tracing was superimposed onto the 100x viewing field for neuron counting. The software randomly sampled predefined counting frames within each outline, allowing for an unbiased sampling of each SNpc section. Total neuron counts were estimated by factoring in the number of TH+ nuclei, size of the SNpc section, and thickness of the counting region. Further details on counting methods have been published previously^95^.

### HPLC analysis

Mouse brain tissue was dissected, weighed, and homogenized in perchloric (0.3 N) acid for the HPLC/ED analysis. The HPLC system consisted of a solvent-delivery system (model 582 pump; ESA), an autosampler (model 542), and a coulometric electrochemical detector (Coulochem III; ESA). The guard cell was set to 350 mV. Electrodes 1 and 2 were adjusted to 150 and 250 mV, respectively. Chromatographic separations were performed on an MD-150, 3.2-m column, and the entire system was run at an ambient temperature. The mobile phase consisted of 85 mM sodium phosphate, 2 mM 1-octanesulfonic acid, 75 mM disodium EDTA, 0.02% triethylamine, and 13% acetonitrile (vol/vol). The pH of the mobile phase was adjusted to 4.85 with sodium hydroxide. These solutions were prepared in HPLC-grade water, filtered through a 0.22-m membrane under vacuum, and pumped at a rate of 0.6 mL/min, producing a background pressure of 181 bars. Samples were further identified by spiking with external monoamine standards; the retention times (in min) of the monoamine standards and co-eluting peaks of samples were recorded. The concentrations of monoamines in the unknown samples were quantified by comparing the peak areas with those of the external monoamine standard chromatograms. All HPLC analysis was performed at Vanderbilt University.

### MAO-B enzyme activity

SNpc and striatal protein from *Gucy2c*^+/+^ and *Gucy2c*^−/−^ mice was homogenized in 200 uL M-PER supplemented with protease inhibitor on ice. Ten μL of the sample was immediately used in the Monoamine Oxidase Enzyme Activity kit (Sigma) in the presence of provided Clorgyline to inhibit MAO-A and isolate MAO-B activity. The protocol followed manufacturer instructions.

Results were normalized to individual sample protein content determined by the BCA assay.

### Messenger RNA isolation, DNA isolation, and qRT-PCR

Messenger RNA isolation was performed on tissue using the Rneasy Plus Mini kit (Qiagen, 74034) and stored at −80 °C for further processing. Reverse transcription was performed with TaqMan Reverse Transcription Reagents (Thermo Fisher, N8080234) according to the manufacturer’s instructions. DNA isolation was performed using the QIAamp DNA Micro (Qiagen 56304). Quantitative RT-PCR was performed using Applied Biosystems TaqMan Master Mix (Thermo Fisher) for cDNA, or with PowerUP Sybr Master Mix (Thermo Fisher) for DNA. Primer probes are listed in Supplemental Table 3. Relative expression was calculated as 2^–∆∆Ct^.

### MN9D culture conditions

MN9D neurons were generously provided by Dr. Zigmond (University of Pittsburgh) and were cultured according to modified developer instructions^43^. Briefly, MN9D cells were plated on 1% poly-l-lysine (Sigma) in DMEM/F12 media (Thermo Fisher) supplemented with 10% FBS and 1% penicillin/streptomycin at a density of 30 K/cm^2^. MN9Ds cultured in serum-starved media were grown in identical conditions, with the DMEM/F12 supplemented with 5% rather than 10% FBS for the final 24 h in culture. To differentiate MN9Ds, media was supplemented with 1 mM butyric acid for 4 d^96^. All experiments were performed on differentiated neurons. Cell-permeable 8-pCPT-cGMP (Biolog Life Science Institute, Germany) was used at a concentration of 100 µM for 48 h^97^. MPP+ was used at 100 µM for 24 h^39,98^.

### MN9D experiments

Each data point shown in imaging experiments represents the average between individual neurons within one well. ATP activity was determined as described above. DNA was isolated as described above. MtDNA was determined by normalizing mtDNA-encoded 16S ribosomal RNA to nuclear DNA-encoded beta-2 microglobulin. As MPP+ reduced mtDNA to undetectable levels in a substantial number of MN9D samples, mtDNA is presented as a binary positive or negative value with a threshold of 20% of average baseline expression. The antioxidant capacity of MN9D lysates was determined using the total antioxidant capacity assay kit (Abcam) according to manufacturer instructions. To measure ROS via chloromethyl derivative 2ʹ,7ʹ-dichlorodihydrofluorescein diacetate (CM-H2DCFDA) (Thermo Fisher), MN9Ds were cultured and treated in black, glass-bottomed 96w plates in phenol-free media and incubated with CM-H2DCFDA according to manufacturer instructions. The fluorescent signal was quantified in a plate reader at a 480/520 excitation/emission wavelength. Cells were next fixed in 4% PFA with DAPI for 10 m at 4 °C and washed once with PBS. The fluorescent signal was quantified via a plate reader at a 355/460 excitation/emission wavelength. Each sample is presented as a CM-H2DCFDA signal normalized to a DAPI signal. To determine cell viability by fluorescent alamarBlue^TM^ assay, MN9Ds were cultured and treated in black, glass-bottomed 96w plates (Nalge Nunc International Corporation) in phenol-free media and incubated with alamarBlueTM (Thermo Fisher) according to manufacturer instructions. To measure ΔΨ_m_ using JC-1 dye, MN9Ds were cultured on black 96-well glass-bottomed well plates and incubated with JC-1 (Thermo Fisher) according to manufacturer instructions. The fluorescent signal of live cells was quantified in a plate reader at a 480/520 and a 584/612 excitation/emission wavelength. To determine ΔΨ_m_ using MitoTracker™, mitochondrial mass, and ROS levels, MN9Ds were cultured on glass coverslips and co-incubated with MitoTracker™ Red CMX Ros (Thermo Fisher) and CellROX™ Far Red (Thermo Fisher) according to manufacturer instructions. Stained and fixed neurons were imaged on a confocal microscope. Mitochondrial membrane potential was measured by calculating the mean fluorescent intensity of MitoTracker™ within neuronal cytoplasm using ImageJ software. Mitochondrial mass was calculated by converting the positive MitoTracker™ signal into a binary mask using ImageJ software. The percent area of cytoplasm that has a positive MitoTracker™ signal is shown in Fig. 6f. The script for quantification is available upon request. To measure ROS using CellROX™, the mean fluorescence intensity of CellROX™ was measured within neuronal cytoplasm. Cell death was determined by plating and treating MN9D neurons in a black, glass-bottomed 96w plate (Nalge Nunc International Corporation) in phenol-free media. Live cells were incubated with cell death marker Sytox Green (Thermo Fisher) according to manufacturer instructions. The fluorescent signal was quantified by a plate reader at a 480/520 excitation/emission wavelength. Cells were then washed with PBS and fixed in 4% PFA and DAPI at 4 °C for 10 min. The fluorescent signal of DAPI was quantified by a plate reader at a 390/410 excitation/emission wavelength.

### Statistics

Results are presented as the mean ± the standard error of the mean (SEM), and a *p* value of <0.05 was considered significant. Statistical analysis was performed in GraphPad Prism 9 (Version 9.3.1). In Fig. 1m, RNAscope values were analyzed using a two-tailed *t*-test. In Fig. 1o, cGMP values were analyzed using a two-way ANOVA with a post hoc false discovery rate <0.05. In Fig. 2d, g, i, n, q, values were analyzed using a two-tailed *t*-test. In Fig. 2f, j, k, statistics were calculated using a two-way ANOVA with a post hoc false discovery rate <0.05. In Fig. 3d, e, i, l, statistics were calculated using a one-way ANOVA with a post hoc false discovery rate <0.05. In Fig. 3f, statistics were calculated using a two-way ANOVA with a post hoc false discovery rate <0.05. In Fig. 4a, h, i, o, statistics were calculated using a two-way ANOVA with a post hoc false discovery rate <0.05. In Fig. 4c, f, g, n, statistics were calculated using a two-tailed *t*-test. In Fig. 4j, k, statistics were calculated using a one-way ANOVA with a post hoc false discovery rate <0.05. In Fig. 5b–e, statistics were calculated using a two-tailed *t*-test. In Fig. 5f, statistics were calculated using a two-way ANOVA with a post hoc false discovery rate <0.05. In Fig. 6e–k, statistics were calculated using a one-way ANOVA with a post hoc false discovery rate <0.05.

### Study approval

The Thomas Jefferson University Institutional Animal Care and Use Committee approved all animal protocols and procedures under protocols 01357 and 01892.

### Supplementary information

Supplemental material

Related Manuscript File

## Data Availability

The data supporting the conclusions of this article are included within the article and its additional files. Human midbrain microarray data were downloaded from Gene Expression Omnibus ID [dataset] GEO: GSE42966 on February 17, 2021. GSE42966 was based on the Agilent GPL4133 platform (Agilent-014850 Whole Human Genome Microarray 4x44K G4112F). Data were freely available online via this link, and our analysis did not involve experiments with humans or animals performed by any of the authors. The GEO2R online analysis tool (https:// www.ncbi.nlm.nih.gov/geo/geo2r/) was used to detect gene levels of Th, Gucy2c, and Vmat2 (Fig. 4a). Additional data are available from the corresponding author upon request.

## Code availability

Mean fluorescent intensities and fluorescent puncta were calculated using custom code written in ImageJ (Version 1.54). Statistics were calculated as described in the manuscript using GraphPad Prism software (version 9.5.1). The code is available from the corresponding author upon request.
