# Supplementary material for: GUCY2C signaling limits dopaminergic neuron vulnerability to toxic insults
Source: NPJ Parkinsons Dis. 2024 Apr 13;10:83. doi: 10.1038/s41531-024-00697-z (PMC11016112; doi:10.1038/s41531-024-00697-z)
Supplement: Supplementary file 1 — Supplemental material [file 41531_2024_697_MOESM1_ESM.pdf]

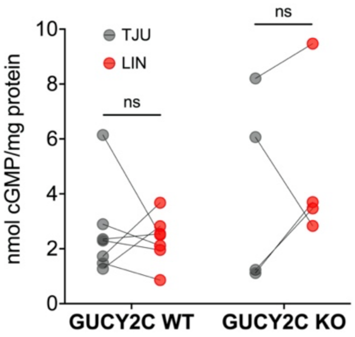

Supplemental figure 1. Stimulating SNpc GUCY2C does not induce extracellular release of cGMP. Guanylyl cyclase C (GUCY2C) agonist linaclotide (LIN) stimulation does not induce extracellular accumulation of cGMP in the *Gucy2*$^{+/+}$ (WT) or *Gucy2c*$^{-/-}$ (KO) substantia nigra pars compacta (SNpc), as determined via cGMP ELISA analysis of supernatant (n=4-7). Statistics were calculated using a two-way ANOVA with a post-hoc false discovery rate <0.05.

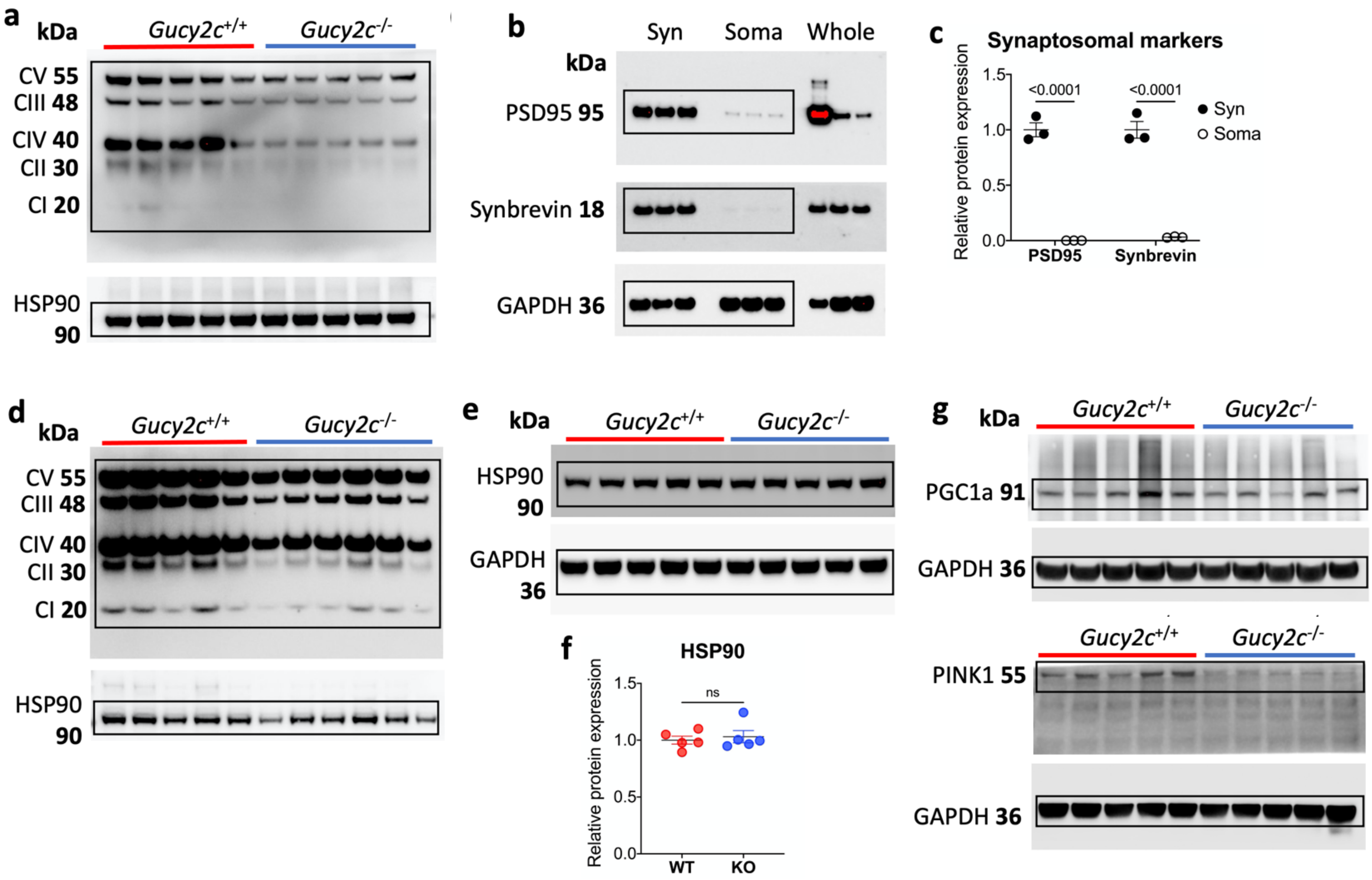

Supplemental figure 2. GUCY2C supports mitochondrial protein expression within the nigrostriatal pathway.

(a-d) *Gucy2c*$^{-/-}$ mice express significantly lower levels of mitochondrial electron transport chain (ETC) proteins in the **(a)** substantia nigra pars compacta (SNpc) and **(b-d)** post-synaptic density 95 (PSD95)+, synaptobrevin (synbrevin)+ striatal synaptosomes (syn) compared to *Gucy2c*$^{+/+}$ (WT) mice (n=5-6). To avoid interference with ETC protein bands, this data is normalized to heat shock protein 90 (HSP90), which is **(e-f)** expressed at comparable levels in *Gucy2c*$^{+/+}$ and *Gucy2c*$^{-/-}$ SNpcs (n=5). **(g)** *Gucy2c*$^{-/-}$ mice express significantly lower levels of peroxisome proliferator-activated receptor gamma coactivator 1-alpha (PGC1a) and PTEN-induced kinase (PINK1) protein in the SNpc as compared to *Gucy2c*$^{+/+}$ mice (n=5). Statistics were calculated using two-way ANOVA with a post-hoc false discovery rate <0.05 (c) and a two-tailed t-test (f). All error bars displayed represent the standard error of the mean (SEM). Molecular weights in kilodaltons (kDa) of each protein analyzed via immunoblots are listed next to the corresponding marker in bolded text.

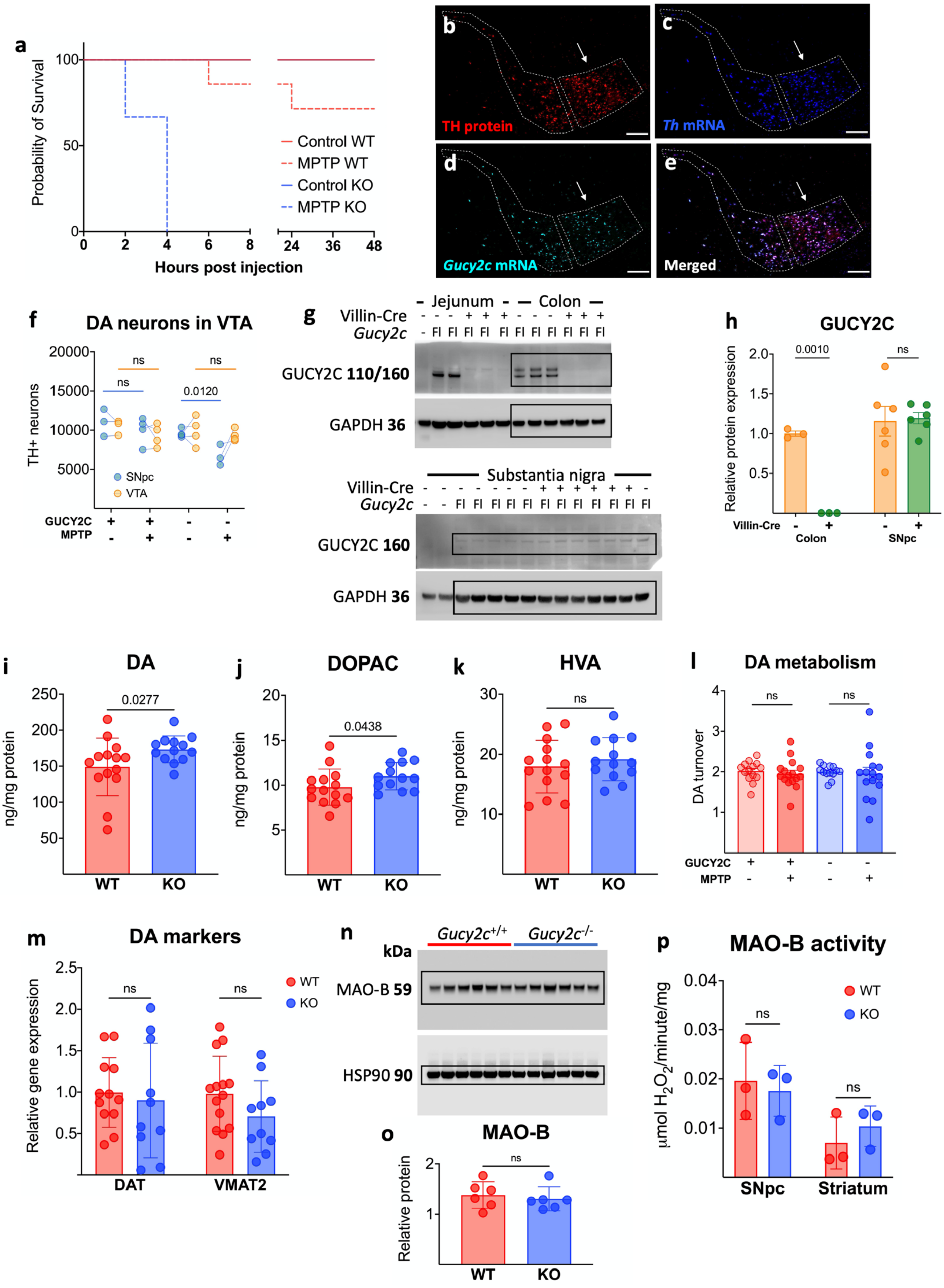

a
Probability of Survival
Hours post injection
Control WT
MPTP WT
Control KO
MPTP KO
b
TH protein
c
Th mRNA
d
Gucy2c mRNA
e
Merged
f
DA neurons in VTA
TH+ neurons
SNpc
VTA
ns
0.0120
GUCY2C
MPTP
g
Jejunum
Colon
Villin-Cre
Gucy2c
GUCY2C 110/160
GAPDH 36
Substantia nigra
GUCY2C 160
h
GUCY2C
Relative protein expression
0.0010
Villin-Cre
Colon
SNpc
i
DA
ng/mg protein
0.0277
WT
KO
j
DOPAC
0.0438
k
HVA
l
DA metabolism
DA turnover
m
DA markers
Relative gene expression
DAT
VMAT2
n
Gucy2c+/+
Gucy2c-/-
kDa
MAO-B 59
HSP90 90
o
MAO-B
Relative protein
p
MAO-B activity
μmol H2O2/minute/mg
SNpc
Striatum

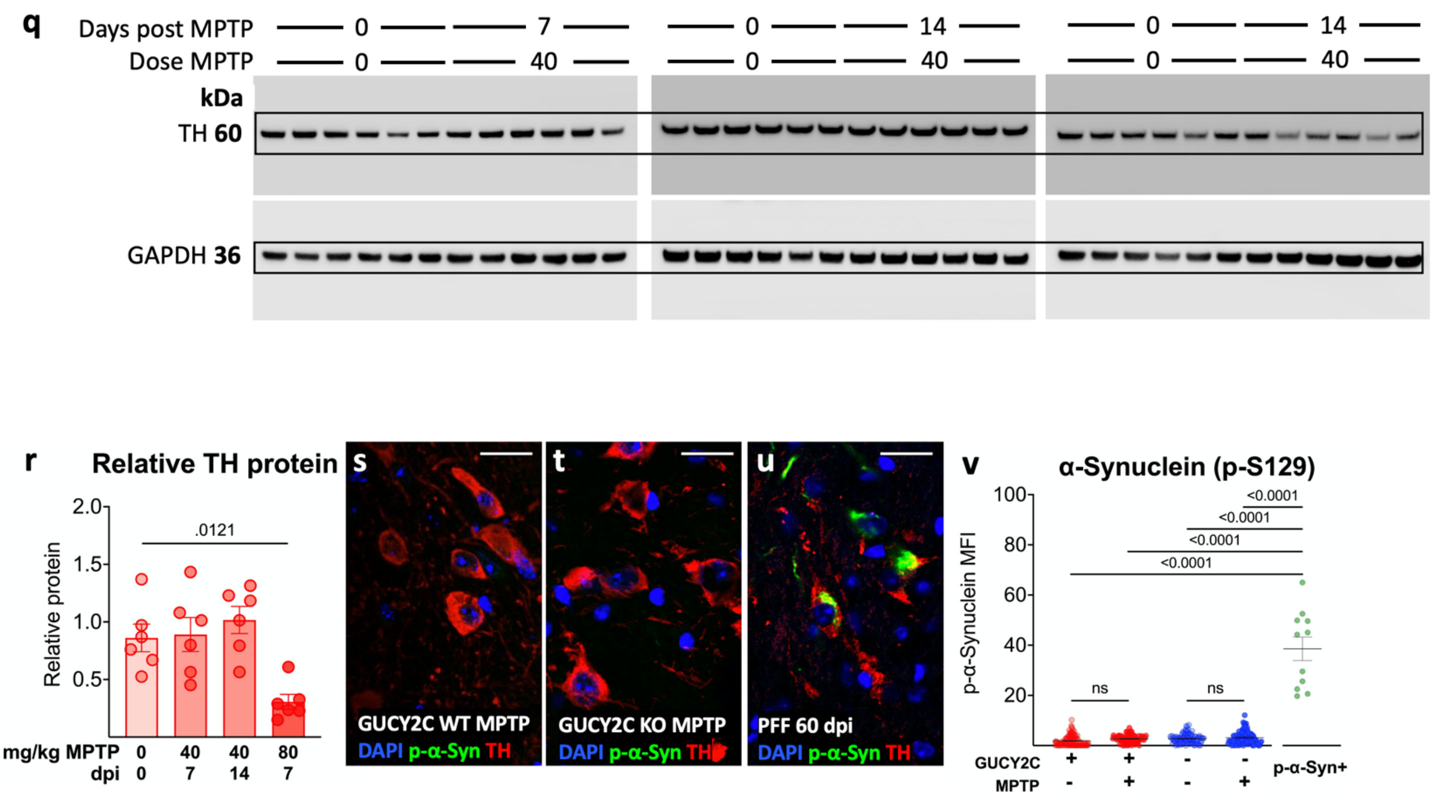

Supplemental figure 3. SNpc GUCY2C mediates vulnerability to MPTP.
(**a**) Intraperitoneal (IP) injections of 4x20 mg/kg of 1-methyl-4-phenyl-1,2,3,6-tetrahydropyridine (MPTP) (standard dosing regimen for acute model) induces lethal toxicity in 100% of *Gucy2c*$^{-/-}$ (KO) mice (n=3-10). **(b-f)** *Gucy2c*$^{+/+}$ (WT) and *Gucy2c*$^{-/-}$ mice do not lose tyrosine hydroxylase (TH)+ neurons in the ventral tegmental area (VTA) following a subtoxic dose of MPTP (n=3-4). VTA is specified with white arrows and scale bars represent 200 µM in images (b-e). **(g-h)** Villin^{cre} x *gucy2c*$^{fl/fl}$ mice selectively lose GUCY2C from the intestine, but not from the substantia nigra pars compacta (SNpc) (n=3-6); [(-), *Gucy2c*$^{-/-}$; (+), *Gucy2c*$^{+/+}$; (Fl), *Gucy2c*$^{fl/fl}$]. **(i-k)** *Gucy2c*$^{-/-}$ mice have higher levels of dopamine (DA) and 3,4-dihydroxyphenylacetic acid (DOPAC), but not homovanillic acid (HVA), at baseline as compared to *Gucy2c*$^{+/+}$ mice (n=13-14). **(l)** DA metabolism is comparable across genotype and MPTP treatment (n=13-14). **(m)** *Gucy2c*$^{+/+}$ and *Gucy2c*$^{-/-}$ mice express comparable levels of dopamine transporter (DAT) and vesicular monoamine transporter 2 (VMAT2) mRNA within TH+ DA neurons (n=10-14, results normalized to TH mRNA). **(n-o)** *Gucy2c*$^{+/+}$ and *Gucy2c*$^{-/-}$ mice express comparable levels of monoamine oxidase-B (MAO-B) protein in the SNpc (n=6) and have **(p)** comparable activity levels of MAO-B in the nigrostriatal pathway (n=3). **(q-r)** Subtoxic MPTP (40 mg/kg) does not induce a loss of TH protein in the *Gucy2c*$^{+/+}$ SNpc at one or two weeks post injection (n=6). Unlike in the preformed fibrils (PFF) mouse model of neurodegeneration, phosphorylated alpha-synuclein (p-α-Syn) inclusions within DA neurons were not induced by subtoxic MPTP in *Gucy2c*$^{+/+}$ or *Gucy2c*$^{-/-}$ mice **(s-v)**. Fluorescence of p-α-Syn+ neurons was quantified from PFF tissue (u-v). Scale bars represent 20 µM (s-u). Statistics were calculated using two-way ANOVA with a post-hoc false discovery rate <0.05 (f, h, m, p), one-way ANOVA with a post-hoc false discovery rate <0.05 (l, r, v), or a two-tailed t-test (i-k, o). All error bars displayed represent the standard error of the mean (SEM). Molecular weights in kilodaltons (kDa) of each protein analyzed via immunoblots are listed next to the corresponding marker in bolded text.

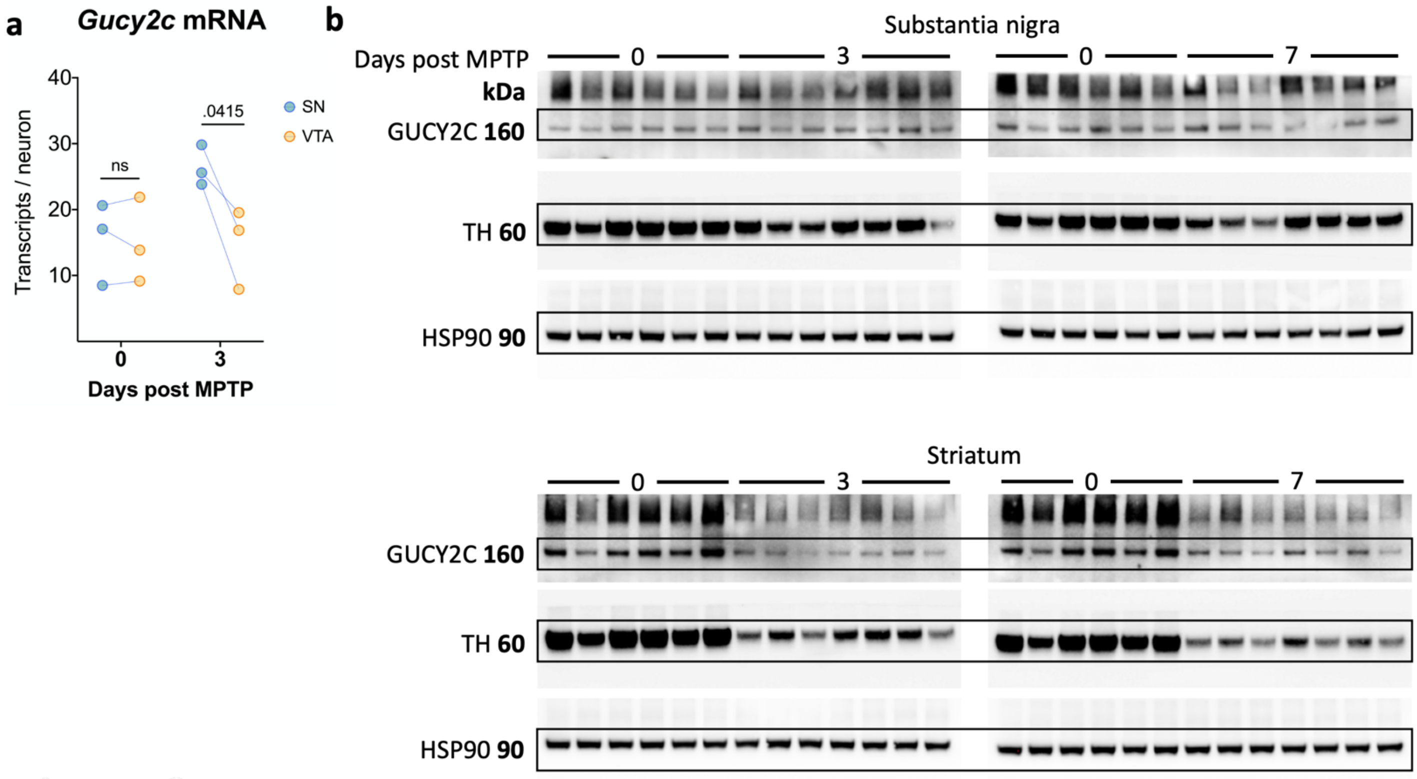

Supplemental figure 4. GUCY2C is overexpressed in pathology.
(a) *Guanylyl cyclase C* (*Gucy2c*) mRNA is upregulated in the *Gucy2c*$^{+/+}$ (WT) substantia nigra pars compacta (SNpc), but not in the ventral tegmental area (VTA), post-1-methyl-4-phenyl-1,2,3,6-tetrahydropyridine (MPTP) (n=3). **(b)** GUCY2C protein is increased relative to tyrosine hydroxylase (TH) in the nigrostriatal pathway following MPTP (n=5-7). Statistics were calculated using a two-way ANOVA with a post-hoc false discovery rate <0.05 (a). Molecular weights in kilodaltons (kDa) of each protein analyzed via immunoblots are listed next to the corresponding marker in bolded text.

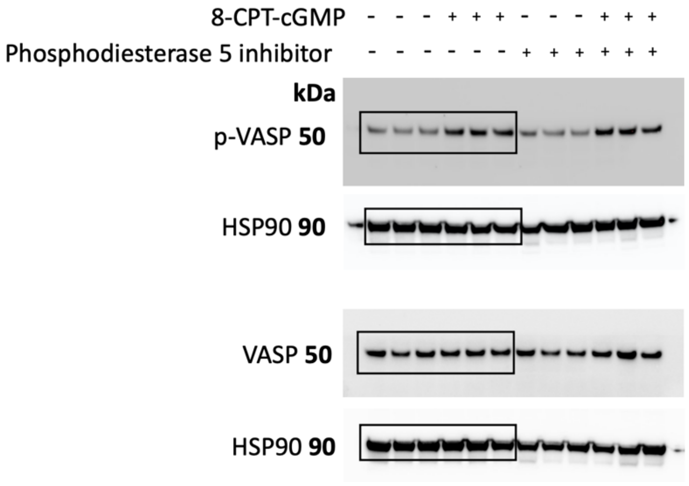

Supplemental figure 5. MN9D neurons are responsive to cGMP signaling.
Cell-permeable cGMP analog 8-(4-chlorophenylthio)guanosine-3', 5'-cyclic monophosphate (8-pCPT-cGMP) increases the phosphorylation of vasodilator-stimulated phosphoprotein (VASP) in MN9D neurons (n=3). All error bars displayed represent the standard error of the mean (SEM). Molecular weights in kilodaltons (kDa) of each protein analyzed via immunoblots are listed next to the corresponding marker in bolded text.

Supplemental table 1. Details of antibodies and RNAscope probes used in immunofluorescence, immunohistochemistry, RNAscope, and immunoblot

Probe	Application	Host	Concentration	Company	Catalog number
TH	IF	Chicken	1:500	Abcam	ab76442
TH	IF, IHC	Rabbit	1:1000	Pel-Freez Biologicals	p40101
Iba1	IF	Rabbit	1:1000	Wako	019-19741
GFAP	IF	Chicken	1:1000	Millipore	AB5541
GUCY2C	IF, IB	Mouse IgG2a	1ug/mL	In-house	N/A
TMEM119	IF	Rabbit	1:1000	Cell Signaling Technology	83308
VDAC1	IF	Rabbit	1:1000	Thermo Fisher	PA1-954A
TOM20	IF	Rabbit	1:1000	Thermo Fisher	MA5-32148
8-oxo-dG	IF	Mouse IgG1	1:100	Abcam	ab145595
beta-III-tubulin	IF	Rabbit	1:1000	Abcam	ab18207
Total OXPHOS Rodent WB cocktail	IB	Mouse IgG1	1:250	Abcam	ab110413
PGC1a	IB	Rabbit	1:1000	Novus	NBP1-04676SS
PINK1	IB	Rabbit	1:1000	Thermo Fisher	PA1-4515
pVASP ser239	IB	Rabbit	1:1000	Cell Signaling Technology	3114
VASP	IB	Rabbit	1:1000	Cell Signaling Technology	3132
MAO-B	IB	Rabbit	1:1000	Millipore	ST1582
pAlpha-synuclein ser129	IF	Rabbit	1:500	Abcam	ab51253
**Probe**	**Application**	**Channel**	**Concentration**	**Company**	**Catalog number**
GUCY2C	RNAscope	C1	As supplied	ACD	436591
TH	RNAscope	C2	1:50	ACD	317621-C2
PINK1	RNAscope	C3	1:50	ACD	524081-C3
Opal 520	RNAscope	520	1:1000	Akoya	FP1487001KT
Opal 690	RNAscope	690	1:1000	Akoya	FP1497001KT

IF = immunofluorescence, IHC = immunohistochemistry, IB = immunoblot

Supplemental table 2. Seahorse experiment inhibitor information

Inhibitor	Working concentration	Volume loaded into port
Oligomycin	4 µM	56 µL
FCCP	1 µM	62 µL
Rotenone /Antimycin A	1 µM	69 µL

FCCP = Carbonyl cyanide-4 (trifluoromethoxy) phenylhydrazone

Supplemental table 3. qPCR primer probe information

Probe	Sequence/ Catalog number	Company	Reagents
msGUCY2C	Mm01267705_m1	ThermoFisher	Taqman
msTH	Mm00447557_m1	ThermoFisher	Taqman
msGAPDH	Mm99999915_g1	ThermoFisher	Taqman
ms16SR FWD	CCGCAAGGGAAAGATGAAAGAC	EuroFins	PowerUP Syber Green
ms16SR REV	TCGTTTGGTTTCGGGGTTTC	EuroFins	PowerUP Syber Green
msB2M FWD	ATGGGAAGCCGAACATACTG	EuroFins	PowerUP Syber Green
msB2M REV	CAGTCTCAGTGGGGGTGAAT	EuroFins	PowerUP Syber Green

ms = mouse, 16SR = 16S ribosomal RNA (mitochondrial-encoded gene), B2M = beta-2-microglobulin (nuclear-encoded gene)
