# Supplementary material for: GUCY2C signaling limits dopaminergic neuron vulnerability to toxic insults
Source: NPJ Parkinsons Dis. 2024 Apr 13;10:83. doi: 10.1038/s41531-024-00697-z (PMC11016112; doi:10.1038/s41531-024-00697-z)
Supplement: Supplementary file 2 — Related Manuscript File [file 41531_2024_697_MOESM2_ESM.pdf]

nature portfolio

Corresponding author(s): Scott Waldman, MD/PhD

Last updated by author(s): Mar 13, 2024

Reporting Summary

Nature Portfolio wishes to improve the reproducibility of the work that we publish. This form provides structure for consistency and transparency in reporting. For further information on Nature Portfolio policies, see our Editorial Policies and the Editorial Policy Checklist.

Statistics

For all statistical analyses, confirm that the following items are present in the figure legend, table legend, main text, or Methods section.

n/a	Confirmed	
☐	☒	The exact sample size (*n*) for each experimental group/condition, given as a discrete number and unit of measurement
☐	☒	A statement on whether measurements were taken from distinct samples or whether the same sample was measured repeatedly
☐	☒	The statistical test(s) used AND whether they are one- or two-sided<br>*Only common tests should be described solely by name; describe more complex techniques in the Methods section.*
☐	☒	A description of all covariates tested
☐	☒	A description of any assumptions or corrections, such as tests of normality and adjustment for multiple comparisons
☐	☒	A full description of the statistical parameters including central tendency (e.g. means) or other basic estimates (e.g. regression coefficient) AND variation (e.g. standard deviation) or associated estimates of uncertainty (e.g. confidence intervals)
☐	☒	For null hypothesis testing, the test statistic (e.g. *F*, *t*, *r*) with confidence intervals, effect sizes, degrees of freedom and *P* value noted<br>*Give P values as exact values whenever suitable.*
☒	☐	For Bayesian analysis, information on the choice of priors and Markov chain Monte Carlo settings
☐	☒	For hierarchical and complex designs, identification of the appropriate level for tests and full reporting of outcomes
☐	☒	Estimates of effect sizes (e.g. Cohen's *d*, Pearson's *r*), indicating how they were calculated

Our web collection on statistics for biologists contains articles on many of the points above.

Software and code

Policy information about availability of computer code

Data collection	Human midbrain microarray data were downloaded from Gene Expression Omnibus ID [dataset] GEO: GSE42966 on February 17, 2021. GSE42966 was based on the Agilent GPL4133 platform (Agilent-014850 Whole Human Genome Microarray 4x44K G4112F). Data were freely available online, and our analysis did not involve experiments with humans or animals performed by any of the authors. The GEO2R online analysis tool (https://www.ncbi.nlm.nih.gov/geo/geo2r/) was used to detect gene levels of Th, Gucy2c, and Vmat2 (Fig. 4a).<br><br>TH+ neurons were visualized using a 100×, 1.3 numerical aperture objective (Olympus, Center Valley, PA) on a BX51 microscope (Olympus) with a MAC5000 motorized XYZ axis computer-controlled stage and a CX9000 CCD video camera (MicroBrightField).
Data analysis	Neurons were counted using a fractionator-sampling design in morphometry and design-based stereology software package, StereoInvestigator, (version 7.0; MicroBrightField, Colchester, VT, USA).<br><br>Mean fluorescent intensities and fluorescent punctae were calculated using custom code written in ImageJ (Version 1.54). Code is available upon request.<br><br>Statistics were calculated as described in the manuscript using GraphPad Prism software (version 9.5.1).

For manuscripts utilizing custom algorithms or software that are central to the research but not yet described in published literature, software must be made available to editors and reviewers. We strongly encourage code deposition in a community repository (e.g. GitHub). See the Nature Portfolio guidelines for submitting code & software for further information.

Data

Policy information about availability of data

All manuscripts must include a data availability statement. This statement should provide the following information, where applicable:

- Accession codes, unique identifiers, or web links for publicly available datasets
- A description of any restrictions on data availability
- For clinical datasets or third party data, please ensure that the statement adheres to our policy

ImageJ scripts used to calculate MFIs and punctae within DA neurons are available upon request. Human midbrain microarray data is freely available to download from GEO using accession number GSE42966.

Research involving human participants, their data, or biological material

Policy information about studies with human participants or human data. See also policy information about sex, gender (identity/presentation), and sexual orientation and race, ethnicity and racism.

Reporting on sex and gender	N/A
Reporting on race, ethnicity, or other socially relevant groupings	N/A
Population characteristics	N/A
Recruitment	N/A
Ethics oversight	N/A

Note that full information on the approval of the study protocol must also be provided in the manuscript.

Field-specific reporting

Please select the one below that is the best fit for your research. If you are not sure, read the appropriate sections before making your selection.

☒ Life sciences ☐ Behavioural & social sciences ☐ Ecological, evolutionary & environmental sciences

For a reference copy of the document with all sections, see nature.com/documents/nr-reporting-summary-flat.pdf

Life sciences study design

All studies must disclose on these points even when the disclosure is negative.

Sample size	Sample sizes were determined using pilot studies and performing power analyses. Observed effect sizes, means, and standard deviations were used to calculate appropriate sample sizes, with a power of 0.8 and a significance level of .05.
Data exclusions	Points with high absolute Z-scores (above two standard deviations from the group mean) were considered outliers and excluded from results.
Replication	Experiments were repeated individually, and further validated with orthogonal methods to verify the reported findings.
Randomization	After controlling for genotype, sex, and age, mice were randomly assigned to experimental conditions. Each mouse experiment conducted had age- and sex-matched littermates across every condition.
Blinding	Investigators were blinded during experiments and data collection. In mouse experiments, mice were randomly assigned numbers, which were decoded after data analyses had been completed.

Reporting for specific materials, systems and methods

We require information from authors about some types of materials, experimental systems and methods used in many studies. Here, indicate whether each material, system or method listed is relevant to your study. If you are not sure if a list item applies to your research, read the appropriate section before selecting a response.

Materials & experimental systems

n/a	Involved in the study
☐	☒ Antibodies
☐	☒ Eukaryotic cell lines
☒	☐ Palaeontology and archaeology
☐	☒ Animals and other organisms
☒	☐ Clinical data
☒	☐ Dual use research of concern
☒	☐ Plants

Methods

n/a	Involved in the study
☒	☐ ChIP-seq
☒	☐ Flow cytometry
☒	☐ MRI-based neuroimaging

Antibodies

Antibodies used

TH Chicken 1:500 Abcam ab76442
TH Rabbit 1:1000 Pel-Freez Biologicals p40101
Iba1 Rabbit 1:1000 Wako 019-19741
GFAP Chicken 1:1000 Millipore AB5541
GUCY2C Mouse IgG2a 1ug/mL In-house N/A
TMEM119 Rabbit 1:1000 Cell Signaling Technology 83308
VDAC1 Rabbit 1:1000 Thermo Fisher PA1-954A
TOM20 Rabbit 1:1000 Thermo Fisher MA5-32148
8-oxo-dG Mouse IgG1 1:100 Abcam ab145595
beta-III-tubulin Rabbit 1:1000 Abcam ab18207
"Total OXPHOS
Rodent WB cocktail" Mouse IgG1 1:250 Abcam ab110413
PGC1a Rabbit 1:1000 Novus NBP1-04676SS
PINK1 Rabbit 1:1000 Thermo Fisher PA1-4515
pVASP ser239 Rabbit 1:1000 Cell Signaling Technology 3114
VASP Rabbit 1:1000 Cell Signaling Technology 3132
MAO-B Rabbit 1:1000 Millipore ST1582
pAlpha-synuclein ser129 Rabbit 1:500 Abcam ab51253
GUCY2C C1 As supplied ACD 436591
TH C2 1:50 ACD 317621-C2
PINK1 C3 1:50 ACD 524081-C3
Opal 520 520 1:1000 Akoya FP1487001KT
Opal 690 690 1:1000 Akoya FP1497001KT
TH Chicken 1:500 Abcam ab76442
TH Rabbit 1:1000 Pel-Freez Biologicals p40101
Iba1 Rabbit 1:1000 Wako 019-19741
GFAP Chicken 1:1000 Millipore AB5541
GUCY2C Mouse IgG2a 1ug/mL In-house N/A
TMEM119 Rabbit 1:1000 Cell Signaling Technology 83308
VDAC1 Rabbit 1:1000 Thermo Fisher PA1-954A
TOM20 Rabbit 1:1000 Thermo Fisher MA5-32148
8-oxo-dG Mouse IgG1 1:100 Abcam ab145595
beta-III-tubulin Rabbit 1:1000 Abcam ab18207
"Total OXPHOS
Rodent WB cocktail" Mouse IgG1 1:250 Abcam ab110413
PGC1a Rabbit 1:1000 Novus NBP1-04676SS
PINK1 Rabbit 1:1000 Thermo Fisher PA1-4515
pVASP ser239 Rabbit 1:1000 Cell Signaling Technology 3114
VASP Rabbit 1:1000 Cell Signaling Technology 3132
MAO-B Rabbit 1:1000 Millipore ST1582
pAlpha-synuclein ser129 Rabbit 1:500 Abcam ab51253

Validation

TH Abcam ab76442 Company validation - positive controls; PC12 cell lysate; Rat glial tumor cell line; Rat cerebral cortex; Mouse cerebral cortex; SH-SY5Y.
TH Pel-Freez Biologicals p40101 Company validation - positive controls; rat caudate lysate showing specific immunolabeling
Iba1 Wako 019-19741 Company validation - Antibody specificity was demonstrated by detection of differential basal expression of the target across cell lines tested owing to their inherent genetic constitution. Relative expression of IBA1 was observed in THP-1 in comparison to A-431, HeLa and MCF7 using Anti-IBA1 Polyclonal Antibody
GFAP Millipore AB5541 Company validation - Rat brain or cerebellum used as positive controls
GUCY2C In-house N/A We validated the specificity of our in-house anti-GUCY2C using positive samples such as intestine, and negative samples such as liver and Gucy2c knockout intestine.
TMEM119 Cell Signaling Technology 83308 Company validation - anti-TMEM119 specifically stained Iba1+ microglia with both ramified and amoeboid morphologies
VDAC1 Thermo Fisher PA1-954A Company validation - This antibody was verified by Knockdown to ensure that the antibody binds to the antigen stated.
TOM20 Thermo Fisher MA5-32148 Company validation - This antibody was verified by Knockdown to ensure that the antibody binds to the antigen stated.
8-oxo-dG Abcam ab145595 Verified in multiple publications, such as in PMID 19412858.

	beta-III-tubulin Abcam ab18207 Company validation via knockout<br>"Total OXPHOS<br>Rodent WB cocktail" Abcam ab110413 Company validation - each subunit validated using positive controls<br>PGC1a Novus NBP1-04676SS Company validation via knockout<br>PINK1 Thermo Fisher PA1-4515 Verified in multiple publications, such as PMID 35954197.<br>pVASP ser239 Cell Signaling Technology 3114 Verified in multiple publications, such as PMID 37710073.<br>VASP Cell Signaling Technology 3132 Verified in multiple publications, such as PMID 37138780.<br>MAO-B Millipore ST1582 Company validation using positive controls<br>pAlpha-synuclein ser129 Abcam ab51253 Company validation using positive controls, with and without phosphatase

Eukaryotic cell lines

Policy information about cell lines and Sex and Gender in Research

Cell line source(s)	Although the MN9D cell line that we used was originally provided from a collaborating laboratory (referenced in the manuscript), these cells are now commercially available through EMD Millipore (catalog no. SCC281). These immortalized dopaminergic neurons were originally generated by fusion between rostral mesencephalic neurons from a 14-day-old C57BL/6J mouse embryo and N18TG2 neuroblastoma cells.
Authentication	MN9D neurons were analyzed for dopaminergic neuronal markers, prior to and following a standard differentiation protocol.
Mycoplasma contamination	MN9D cells tested negative for mycoplasma contamination.
Commonly misidentified lines<br>(See ICLAC register)	N/A

Animals and other research organisms

Policy information about studies involving animals; ARRIVE guidelines recommended for reporting animal research, and Sex and Gender in Research

Laboratory animals	All mice used in this study were aged 3-6 months and were on a C57Bl6/J background. We used our laboratory-generated Gucy2c wildtype vs knockout strain (all studies used age- and sex-matched littermates), with and without a TH-GFP knock-in, as described in our manuscript.
Wild animals	The study did not involve samples from wild animals.
Reporting on sex	We controlled for sex in all experiments. Males and females from both genotypes were evenly distributed across treatment groups. Within each genotype, there was no differential response between male and female mice to subtoxic MPTP across our measurements, including surviving dopaminergic neurons, HPLC measurements of dopamine, DOPAC, and HVA, or gliosis. We also did not observe a baseline difference between sex in mitochondrial protein expression, oxygen consumption, or ATP levels. Below, please find the sexes of mice used for each experiment.<br><br>Study: MPTP-induced DA neuron loss 21 wildtype males 23 wildtype females 14 knockout males 12 knockout females<br>Study: MPTP-induced DA, DOPAC, and HVA loss 17 wildtype males 13 wildtype females 15 knockout males 13 knockout females<br>Study: MPTP-induced gliosis 4 wildtype males 4 wildtype females 3 knockout males 5 knockout females<br>Study: VDAC and TOM20 expression 6 wildtype males 5 wildtype females 7 knockout males 4 knockout females<br>Study: Mitochondria ETC, PINK1, and PGC1a protein expression 3 wildtype males 2 wildtype females 2 knockout males 3 knockout females<br>Study: ATP levels 4 wildtype males 2 wildtype females 3 knockout males 3 knockout females<br>Study: Pink1 mRNA transcripts 3 wildtype males 4 wildtype females 3 knockout males 4 knockout females<br>Study: 8-oxo-dG 2 wildtype males 2 wildtype females 2 knockout males 2 knockout females<br>Study: Gucy2c mRNA in wildtype vs knockout SNpc 2 wildtype males 2 wildtype females 2 knockout males 2 knockout females<br>Study: MPTP-upregulation of Gucy2c mRNA qPCR 11 wildtype males 14 wildtype females<br>Study: MPTP-induced upregulation of Gucy2c protein (immunoblot) 9 wildtype males 8 wildtype females<br>Study: MPTP-induced upregulation of cGMP production 7 wildtype males 7 wildtype females<br>Study: MPTP-induced DA neuron loss in Villin-Cre mice 7 Villin-Cre+ males 5 Villin-Cre+ females 6 Villin-Cre- females 6 Villin-Cre- females
Field-collected samples	The study did not involve samples collected from the field.
Ethics oversight	IACUC approved all breeding, maintenance, and experimental procedures used in this study.

Note that full information on the approval of the study protocol must also be provided in the manuscript.

Plants

Seed stocks	The study did not involve seed stocks.
Novel plant genotypes	The study did not involve novel plant genotypes.
Authentication	N/A
